# Discharge and Role of Acetylcholine Pontomesencephalic Neurons in Cortical Activity and Sleep-Wake States Examined by Optogenetics and Juxtacellular Recording in Mice

**DOI:** 10.1523/ENEURO.0270-18.2018

**Published:** 2018-09-13

**Authors:** Youssouf Cissé, Hanieh Toossi, Masaru Ishibashi, Lynda Mainville, Christopher S. Leonard, Antoine Adamantidis, Barbara E. Jones

**Affiliations:** 1 Department of Neurology and Neurosurgery, Montreal Neurological Institute, McGill University, Montreal, Quebec H3A 2B4, Canada; 2 Department of Physiology, New York Medical College, Valhalla, NY 10595; 3 Department of Biomedical Research and Department of Neurology, Zentrum Für Experimentelle Neurologie, Inselspital University Hospital Bern, University of Bern, Bern, CH-3010, Switzerland

**Keywords:** γ, paradoxical sleep, REM sleep, slow wave sleep, θ, waking

## Abstract

Acetylcholine (ACh) neurons in the pontomesencephalic tegmentum (PMT) are thought to play an important role in promoting cortical activation with waking (W) and paradoxical sleep [PS; or rapid eye movement (REM)], but have yet to be proven to do so by selective stimulation and simultaneous recording of identified ACh neurons. Here, we employed optogenetics combined with juxtacellular recording and labeling of neurons in transgenic (TG) mice expressing ChR2 in choline acetyltransferase (ChAT)-synthesizing neurons. We established *in vitro* then *in vivo* in anesthetized (A) and unanesthetized (UA), head-fixed mice that photostimulation elicited a spike with short latency in neurons which could be identified by immunohistochemical staining as ACh neurons within the laterodorsal (LDT)/sublaterodorsal (SubLDT) and pedunculopontine tegmental (PPT) nuclei. Continuous light pulse stimulation during sleep evoked tonic spiking by ACh neurons that elicited a shift from irregular slow wave activity to rhythmic θ and enhanced γ activity on the cortex without behavioral arousal. With θ frequency rhythmic light pulse stimulation, ACh neurons discharged in bursts that occurred in synchrony with evoked cortical θ. During natural sleep-wake states, they were virtually silent during slow wave sleep (SWS), discharged in bursts during PS and discharged tonically during W. Yet, their bursting during PS was not rhythmic or synchronized with cortical θ but associated with phasic whisker movements. We conclude that ACh PMT neurons promote θ and γ cortical activity during W and PS by their tonic or phasic discharge through release of ACh onto local neurons within the PMT and/or more distant targets in the hypothalamus and thalamus.

## Visual Abstract

**Figure:**
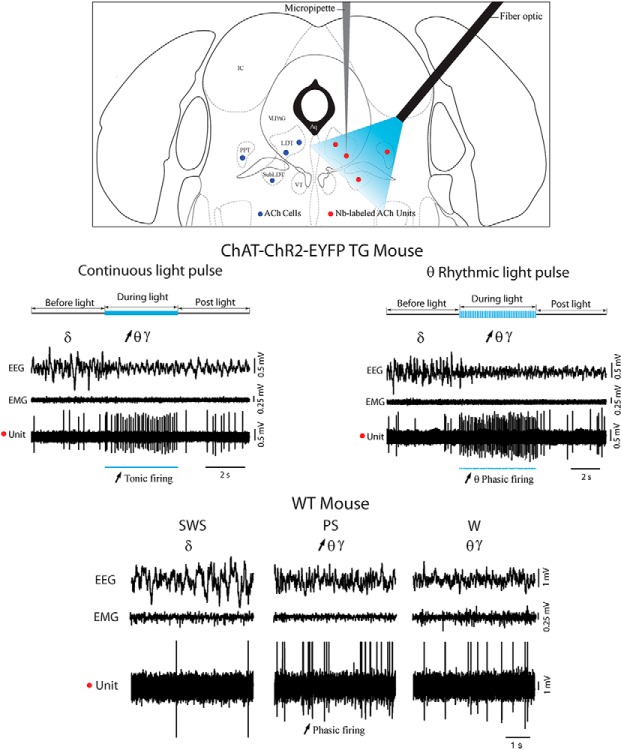

## Significance Statement

By selectively stimulating and recording acetylcholine (ACh) neurons in the pontomesencephalic tegmentum (PMT), we show that evoked tonic or θ rhythmic phasic firing by ACh neurons attenuates slow wave activity and stimulates θ and γ activity on the cerebral cortex without eliciting behavioral arousal from sleep. During natural sleeping-waking, ACh neurons are virtually silent during slow wave sleep (SWS), discharge in bursts during paradoxical sleep (PS) or rapid eye movement (REM) and discharge in a tonic manner during waking (W). We conclude that by tonic or burst discharge during W and PS, ACh PMT neurons promote θ with γ activities that are known to be important for memory along with other cortical processes during these states.

## Introduction

Very important roles have been attributed to the cholinergic neurons of the pontomesencephalic tegmentum (PMT) in the regulation of cortical activity and sleep-wake states (for review, see Jones, 2011, 2017; Brown et al., 2012; Scammell et al., 2017). In early studies, the cholinergic neurons were believed to comprise the ascending reticular activating system that was considered essential for the maintenance of cortical activation and waking (W). In later studies, they were also proposed to serve as the executive neurons for the generation of rapid eye movement (REM) or paradoxical sleep (PS), which is characterized by cortical activation accompanied by postural muscle atonia. Many lines of evidence from pharmacological to immunohistochemical with lesion, c-Fos expression and most recently electrophysiological studies have substantiated a key role for these cholinergic neurons in W and PS (Baghdoyan et al., 1984b; Webster and Jones, 1988; Maloney et al., 1999; Boucetta et al., 2014), yet other lines have called into question their importance (Luppi et al., 2006; Saper and Fuller, 2017). Clearly additional experiments permitting the specific activation and recording of cholinergic neurons are necessary for further understanding the discharge and role of these neurons in cortical activation and sleep-wake states.

Many early studies demonstrated that local microinjections of cholinergic agonists, particularly carbachol, into the brainstem could elicit cortical activation with W or PS (George et al., 1964; Baghdoyan et al., 1984a). Injected into the oral pontine reticular formation, carbachol could elicit θ oscillations, which represent a cardinal component of PS as well as active/attentive W and serve in memory consolidation during these states (Vertes et al., 1993; Boyce et al., 2016). In these same regions, carbachol evoked full PS with muscle atonia that could be blocked by the muscarinic antagonist, atropine (Gnadt and Pegram, 1986). Moreover, microinjection of the acetylcholinesterase inhibitors into the pons could facilitate PS, indicating that endogenously released acetylcholine (ACh) could promote PS (Baghdoyan et al., 1984b). Proof of the role of the PMT ACh neurons in this process, however, has awaited demonstration of their specific activation that has become possible with the application of optogenetics and chemogenetics. Two recent studies have employed these techniques. The first, using optogenetics showed that photostimulation of the PMT ACh neurons could enhance REM sleep (Van Dort et al., 2015). The second, using chemogenetics with DREADD (designer receptor exclusively activated by designer drugs) showed that prolonged pharmacological stimulation of the PMT ACh neurons did not significantly alter amounts of either W or REM sleep, but attenuated the slow wave activity of slow wave sleep (SWS; Kroeger et al., 2017). Yet both of these studies suffered limitations related to the expression of the probes (see Discussion), and neither recorded from the cholinergic neurons *in vivo* to know their discharge in response to the opto- or chemogenetic stimulation and thus in direct relation to electroencephalographic (EEG) and sleep-wake state changes.

Multiple early studies involved recording of neurons in the region of PMT cholinergic neurons in association with EEG activity and sleep-wake states (El Mansari et al., 1989; Sakai et al., 1990; Steriade et al., 1990a,b; Sakai and Koyama, 1996). Evidence was presented that putative cholinergic neurons in the region of the laterodorsal (LDT) and sublaterodorsal (SubLDT) and pedunculopontine tegmental (PPT) nuclei would discharge in association with cortical activation during W and PS. However, it is only recently that by application of juxtacellular recording and labeling combined with immunohistochemical identification of the recorded neuron that the discharge of identified ACh neurons within the LDT/SubLDT and medial PPT nuclei was possible in rats (Boucetta et al., 2014). Nonetheless, this technique is extremely difficult and only permits one unit per side per animal to be labeled to have certainty as to the cell recorded and subsequently identified as the neurobiotin (Nb)-labeled neuron. With the development of optogenetics, it has become possible to identify neurotransmitter specific neurons, including ACh neurons, by photostimulation *in vivo* (Zhao et al., 2011). We thus used optogenetics to record from photo-identified ACh neurons first *in vitro* then in anesthetized (A) and unanesthetized (UA) head-fixed, naturally sleeping-waking transgenic (TG) mice. We combined the optogenetics with juxtacellular labeling to confirm that the photo-identified neurons were ACh neurons and to localize the recorded neurons within the PMT. We were thereby able to examine with the temporal precision of optogenetic stimulation, the evoked discharge of confirmed PMT ACh neurons in relation to evoked changes in EEG activity and sleep-wake state in TG mice. We further examined their discharge during natural sleep-wake states to compare their natural discharge profiles in relation to natural EEG activity and sleep-wake states in wild-type (WT) mice and thereby fully understand their role in the sleep-waking cycle.

## Materials and Methods

### Animals

Recording experiments were performed *in vitro* and *in vivo* using TG mice, which were bred to express channelrhodopsin-2 (ChR2) fused to enhanced yellow fluorescent protein (EYFP) in choline acetyltransferase (ChAT) neurons (abbreviated as ChAT-ChR2-EYFP mice), along with WT C57BL/6 mice of both sexes. The parental strains of the TG mice were obtained from The Jackson Laboratory (http://jax.org/strain/012569, for Cre-inducible ChR2-EYFP and http://jax.org/strain/006410, for ChAT-IRES-Cre mice). The WT mice came from Charles River (http://criver.com/products-services/basic-research/find-a-model/c57bl-6n-mouse ). Development and experimental use of ChAT-ChR2-EYFP TG mice were previously shown by others to be effective for the electrophysiological and behavioral study of central cholinergic neurons (Zhao et al., 2011; Hedrick et al., 2016). For the *in vitro* studies, all procedures complied with NIH guidelines and were approved by New York Medical College Institutional Animal Care and Use Committee. For the *in vivo* studies, all procedures complied with the Canadian Institutes of Health Research guidelines and were approved by the McGill University Animal Care Committee and the Canadian Council on Animal Care.

### *In vitro* recording in brain slices

Brain slices (250 μm) were prepared from ChAT-ChR2-EYFP (P15–P16) mice according to previously published methods (Ishibashi et al., 2015). Briefly, slices were cut with a vibratome using ice-cold, oxygenated cutting solution containing 124 mM NaCl, 2.5 mM KCl, 1.2 mM NaH_2_PO_4_, 2.7 mM CaCl_2_, 1.2 mM MgSO_4_, 26 mM NaHCO_3_, and 10 mM dextrose (295–305 mOsm). Slices were then incubated for 10 min in an *N*-methyl-D-glucamine (NMDG) recovery solution (Ting et al., 2014) at room temperature before being rinsed (5×) in cutting solution and transferred to artificial CSF (ACSF) containing 124 mM NaCl, 5 mM KCl, 1.2 mM NaH_2_PO_4_, 2.0 mM CaCl_2_, 1.2 mM MgSO_4_, 26 mM NaHCO_3_, and 10 mM dextrose (295–305 mOsm) at room temperature for storage and use. Whole-cell recordings were obtained using an Axopatch 200B amplifier and borosilicate micropipettes (2–4 MΩ) in slices superfused at 1–2 ml/min with oxygenated ACSF at room temperature. Neurons were visualized using near-infrared DIC optics on a fixed stage microscope (Olympus BX50WI). Given that the intensity of the EYFP was too low for adequate visualization of ChR2-EYFP containing cells, cells were selected according to morphology. The pipette solution contained 144 mM K-gluconate, 3 mM MgCl_2_, 10 mM HEPES, 0.3 mM NaGTP, 4 mM Na_2_ATP (310 mOsm), and bis-fura-2 (50 μM; Invitrogen) for calcium chelation and imaging. Biotinylated Alexa Fluor 594 (25 μM; Invitrogen) was also included in all experiments for subsequent cell identification and localization. All cells reported were located in the LDT. In previous studies, it was determined that ∼76% of LDT ChAT+ neurons contained ChR2-EYFP and that ∼99% of EYFP+ cells were ChAT+ in the LDT of the same ChAT-ChR2-EYFP mice (Ishibashi et al., 2015). It was also established that ACh neurons were generally medium to large in size, according to which such cells were visually selected in the current recordings. Consequently, all cells selected that responded to photostimulation were considered to be presumptive (p)ACh neurons. For photostimulation, a TTL-gated, 473-nm laser (100 mW; SLOC) was coupled to an optic fiber (200 μm in diameter core, 0.22 NA) that was positioned 1.0–1.5 mm above the target region of the slice. Whole-cell data were analyzed using Igor Pro 6 (Wavemetrics). All reported values of Vm were corrected by -15 mV to compensate for the measured liquid junction potentials.

### *In vivo* recording in mice

Mice were housed under a 12/12 h light/dark cycle schedule with lights on from 7:00 A.M. to 7:00 P.M. and had free access to food and water. Adult TG and WT mice (25–30 g) were used for acute recording studies with anesthesia or for chronic recording studies without anesthesia using head-fixation following surgical implantation of a head-fixation post.

For recordings in TG and WT A mice, anesthesia was induced with isoflurane administered within a Plexiglas box (∼5%) and then maintained through a mask (∼2.5%) during surgery. Body temperature was maintained at 36–37°C by a thermostatically controlled heating pad through a rectal thermal probe. The mice were positioned in a stereotaxic frame (David Kopf Instruments) for both surgery and subsequent recording. To record activity of the EEG, stainless steel screws were placed bilaterally over the retrosplenial cortex 1.0 mm posterior and 0.5–0.65 mm lateral to bregma and unilaterally in the frontal bone as a reference. Two holes were opened in the skull over the region of the lambdoid suture, one for the optic fiber and one for the recording micropipette. Following resection of the dura mater in each hole, the isoflurane was diminished and replaced by urethane (ethyl carbamate, Sigma) using an initial large dose (1 g/kg, i.p.) followed by supplementary small doses (0.1–0.15 g/kg), if necessary as indicated by the presence of a withdrawal response of the limb to pinch. The animal was transferred in the stereotaxic apparatus to the recording chamber where the optic fiber (200 μm, ThorLabs) was inserted at ∼1 mm posterior and ∼3 mm lateral to Lambda and descended at a 40° angle from vertical ∼2.0–2.5 mm from the brain surface. EEG and unit recording then proceeded.

For recordings in the TG and WT UA mice, the animals were first operated for implantation of electrodes and a head-fixation post. For this surgery, anesthesia was induced (5%) and maintained with isoflurane (∼2.5%). As for the TG-A mice, screws were implanted over the retrosplenial cortex and frontal bone for EEG and embedded in acrylic cement into which the metal head-fixation post was also fixed. In the TG-UA and WT-UA mice, silver wire loops were also inserted in the neck muscles for recording of the electromyogram (EMG). The EEG and EMG wires were sent to a connector fixed to the left side of the head. As for the TG-A mice, the skull was cleaned in the region around the lambdoid suture. A hole was drilled for the placement of an indwelling cannula (Plastics One) according to the coordinates of the optic fiber which would subsequently be inserted at the time of recording. Acrylic cement was applied to surround the cannula while leaving a small area of the skull around the lambdoid suture clean but covered for subsequent opening to insert the micropipette at the time of recording. Following recovery from the surgery (∼3–5 d), the mice were introduced to the head-fixation apparatus in which their head post was inserted into a receptacle while their body was comfortably contained within a tube lined with foam rubber. The time of habituation to the head-fixation was gradually increased from ∼30 min up to ∼5 h over a period of 10–15 d until the mice would be quietly awake or asleep during the afternoon period of recording, which is their normal maximal sleeping period. Following complete habituation, the mice were anesthetized again with isoflurane (∼2.5%) for drilling of the hole in the region of the lambdoid suture for insertion of the micropipette. The mouse was returned to his home cage until the next day.

### *In vivo* unit recording and labeling

**Figure 1.**
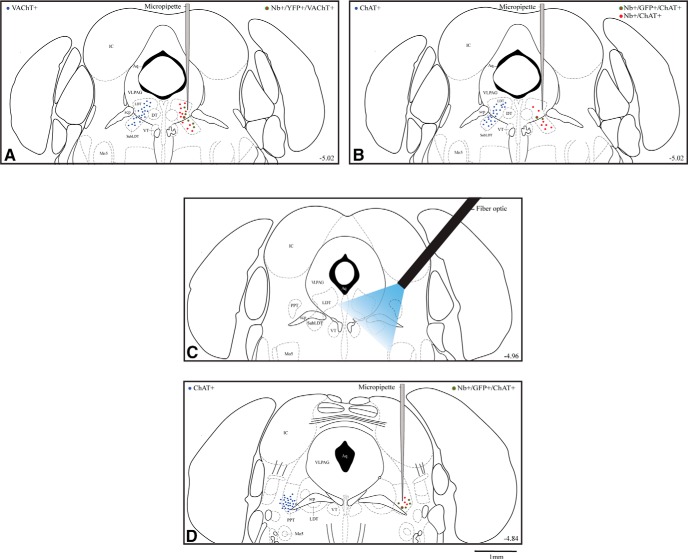
Map of ACh neurons which were recorded and photo-stimulated in ChAT-ChR2-EYFP TG mice. Location of ACh neurons (filled blue circles, left side) which were targeted for recording and photostimulation *in vitro* in brain slices (in LDT) and *in vivo* (in LDT/SubLDT and PPT). From the *in vivo* studies, the approximate location of Nb-labeled ACh neurons (filled red circles) which manifested or not ChR2-EYFP fluorescence (green outline) is shown along with the Nb-filled recording micropipette on the right side of panels ***A***, ***B*** (at ∼-5.02 from bregma) and ***D*** (∼-4.84). The approximate position of the optic fiber is shown in ***C***(∼-4.96). Large symbols indicate the Nb-labeled ACh EYFP stained cells shown in Figure 2. ***A***, In the LDT/SubLDT of A TG mice, the distribution of ACh neurons (identified as VAChT+) is shown on the left and plots of Nb-labeled neurons (Nb+), which were ACh (identified as VAChT+) and manifested ChR2-EYFP (identified as YFP+), on the right side. ***B***, In the LDT/SubLDT of UA TG mice, the distribution of ACh neurons (identified as ChAT+) is shown on the left and plots of Nb-labeled neurons (Nb+), which were ACh (identified as ChAT+) and manifested ChR2-EYFP (identified as GFP+), on the right side. ***C***, Approximate position of the optic fiber and the estimated distribution of blue light (see Materials and Methods) is shown reaching the LDT/SubLDT and a portion of the PPT. ***D***, In the PPT of TG-UA mice, the distribution of ACh neurons (identified as ChAT+) is shown on the left side and plots of Nb-labeled neurons (Nb+), which were ACh (identified as ChAT+) and manifested ChR2-EYFP (identified as GFP+), on the right side. Neurons were plotted onto one atlas level representing the central level for each cell group. Aq, aqueduct of Sylvius; DT, dorsal tegmental nucleus; IC, inferior colliculus; LDT, lateral dorsal tegmental nucleus; Mo5, motor trigeminal nucleus; scp, superior cerebral peduncle; SubLDT, sublateral dorsal tegmental nucleus; VLPAG, ventrolateral periaqueductal gray; VT, ventral tegmental nucleus. Plates adapted from Paxinos and Franklin (2001). Scale bar, 1 mm.

On the day of recording, the TG-UA and WT-UA mice were placed in the head-fixation apparatus. The dura mater in the hole was superfused with lidocaine and incised to allow descent of the micropipette to the LDT/SubLDT or PPT. The TG-A, TG-UA, and WT-UA mice were subsequently transferred within the stereotaxic apparatus to the recording chamber. The optic fiber was lowered at a 40° angle (through brain tissue in the TG-A mice and the indwelling cannula in TG-UA and WT-UA mice) through the inferior colliculus to stop at a position dorsal and lateral to the LDT/SubLDT and PPT (Fig. 1*C*). For recording, a glass micropipette (∼1-μm tip, ∼40 MΩ) filled with ∼5% Nb; Vector Laboratories) in 0.5 M NaCl solution was lowered vertically at ∼1 mm posterior and 0.5–1.3 mm lateral to Lambda using a Kopf micropositioner (Model 660, David Kopf Instruments) to target the LDT/SubLDT or PPT areas (Fig. 1*A*,*B*,*D*). Single units were recorded and labeled using an intracellular amplifier (Neurodata IR-283A, Cygnus Technology). The unit signal was amplified (2000×), digitized at a sampling rate of 8 kHz, and filtered (bandpass filter: 0.3–3 kHz) using a CyberAmp 380 (Molecular Devices) and acquired for online viewing with the Axoscope software (version 10.1, Molecular Devices). The unit was simultaneously recorded with EEG (sampled at 250 Hz and filtered between 0.5 and 100 Hz) and EMG (filtered between 10 and 100 Hz) in TG-UA and WT-UA mice and together with video of the animal’s head in TG-UA mice, using Harmonie software (version 5.2, Stellate).

Photostimulation with blue light (473 nm) was applied through the optic fiber (∼200-μm core diameter, 0.22 NA, Thorlabs) using a laser (Laserglow Technologies, LRS-0473-PFM-00-100-05, >100 mW) connected to a TTL-gated power supply (R471005FX) that was in turn connected to a pulse signal generator (Master 8, AMPI). The laser power for stimulation was set in the TG-A mice to ∼40 mW and in the TG-UA mice to ∼30 mW at the tip, which would correspond to ∼14 and ∼11 mW mm^−2^, respectively, at 1 mm from the tip (https://web.stanford.edu/group/dlab/cgi-bin/graph/chart.php) and the approximate region of the LDT/SubLDT cholinergic cells (Fig. 1). The light intensity used lies within what is considered to be a safe range of ≤75 mW mm^−2^ for *in vivo* experiments (Cardin et al., 2010). During recording, light pulses of 50- or 100-ms duration were delivered every second while searching for responding units in the region of the LDT/SubLDT or PPT. Once a responding unit was identified, the light pulse was reduced to 10–15 ms to test whether the unit emitted a spike within a short light pulse. Units were subsequently examined in association with EEG activity in response to sensory stimulation (tail pinch), a long (∼5 s) continuous light pulse and/or rhythmic light pulses at low θ frequency in TG-A mice (100-ms pulse at 4 Hz for ∼5 s) and a high θ frequency in TG-UA mice (50 ms at 8 Hz for ∼5 s). The response to photostimulation was tested also in WT-UA mice as controls. In some TG-UA animals and WT-UA animals, units together with EEG and EMG were recorded during undisturbed natural sleeping-waking.

Following characterization, units were submitted to juxtacellular labeling using positive current pulses (1–10 nA, 200 ms) delivered for a period ∼2 min (Pinault, 1996). In any one mouse, multiple units were selected for juxtacellular labeling. In the initial experiments in TG-A mice, units were selected during the experiment as to whether they appeared or not to respond to the short (15 ms) light pulse by emitting a spike. However, on analysis some units proved to have not responded during the short light pulse thus including both responding and nonresponding units in the same mouse. In the subsequent studies in the TG-UA mice, units could be more precisely selected for labeling such that Nb-labeled cells within any one mouse would include only responding or nonresponding units and thus allow the collective identification of the units recorded in a mouse as ACh or NonACh.

At the end of the recording session (∼5 h), the TG or WT A mice were administered an overdose of urethane (3 g/kg, i.p.) and the UA mice of sodium pentobarbital (Euthanyl, 100 mg/kg, i.p., Bimeda-MTC Pharmaceuticals) and perfused transcardially with 100 ml of cold saline, followed by 200 ml of cold 3% paraformaldehyde solution for fixation of the brain with euthanasia. The brains were removed, postfixed overnight in the fixative solution and immersed for 2–3 d in 30% sucrose in phosphate buffer for cryoprotection. They were frozen at -50°C and stored at -80°C.

### *In vivo* electrophysiological signal analysis

Simultaneously recorded unit, EEG and EMG signals from TG and WT mice were processed using MATLAB (R2011b, MathWorks) with programs previously developed and described for unit recording in urethane-anesthetized rats (Manns et al., 2000; Boucetta and Jones, 2009) and UA head-fixed rats (Lee et al., 2005; Boucetta et al., 2014). Accordingly, unit average discharge rate, instantaneous firing frequency [the reciprocal of the modal interspike interval (ISI)] and autocorrelation function were calculated together with the EEG power spectrum and unit-to-EEG cross correlated activity from spike-triggered averages (STAs). These measures were computed for the 5-s periods before and during the long continuous and rhythmic light pulse stimulations. In addition, integrated average amplitude of EEG frequency band activity was computed for δ (0–3.5 Hz) and γ (30–58 Hz) along with that of EMG activity (30–100 Hz). These measures were collected for the experiments involving photostimulation in TG mice and also for periods of natural sleep-wake states in TG-UA and WT-UA mice.

### Histochemistry

Brains from the *in vivo* studies were cut in serial sections at 25-μm thickness on a freezing microtome. Sections were collected through the PMT for staining of Nb. They were incubated for 2.5 h in Cy3-conjugated streptavidin (SA-Cy3, 1:1000, catalog #016-160-084, RRID: AB_2337244, Jackson ImmunoResearch), mounted and coverslipped with glycerol for examination under the fluorescence microscope. Sections containing Nb-labeled cells along with other sections through the region of the LDT/SubLDT and PPT were then removed from the slides and processed for immunofluorescent staining. In the TG-A mice, the presence of ChR2-EYFP in neurons was assessed by the native fluorescence of the EYFP, whereas in the TG-UA mice, it was assessed by immunostaining of the EYFP using an antibody to green fluorescent protein (GFP; from rabbit (Rb), 1:3000, catalog #ab290, RRID:AB_303395, Abcam).

For identifying ACh neurons, an antibody for the vesicular transporter protein for ACh (VAChT) was used in the TG-A mice [from goat (Gt), 1:5000, catalog #AB1578, RRID: AB_10000324, Millipore Bioscience Research Reagents or 1:1000, catalog #ABN100, RRID: AB_2630394, Millipore] and one for ChAT in the TG-UA mice (from Gt, 1:500–1:750, catalog #AB144, RRID: AB_11212843, Millipore). Following incubation with the primary antibodies overnight, the sections were rinsed and incubated for 2 h in appropriate combinations of cyanine-conjugated (Cy2 or Cy5) secondary antibodies from donkey (Jackson ImmunoResearch): Cy2-conjugated anti-Rb (1:200; catalog #711-225-152 RRID:AB_2340612) and Cy5-conjugated anti-Gt (1:800, catalog #705-175-147, RRID: AB_2340415, Jackson ImmunoResearch). Finally, sections were rinsed, mounted, and coverslipped with glycerol.

### Fluorescent image analysis

Processed sections were viewed using a Leica DMLB microscope equipped with an x/y/z motorized stage, a digital camera (Orca-R^2^, C10600-10B, Hamamatsu photonics K.K) and fluorescence filters for excitation and emission of Cy2, Cy3, and Cy5 dyes. Following immunostaining of sections containing Nb-labeled cells, each cell was examined for positive or negative fluorescence for EYFP (as native YFP or enhanced anti-GFP immunofluorescence) and for ACh (as VAChT or ChAT immunofluorescence). In addition, one to three sections through the LDT/SubLDT and PPT from each brain were examined for immunostaining to estimate the percentage of ACh neurons which were positive for EYFP and that of EYFP positive neurons which were positive or negative for ACh in the brains of the TG mice. For this purpose, images were acquired and analyzed using StereoInvestigator software (MicroBrightField, MBF) and the Optical Fractionator Probe that permit unbiased, systematic random sampling of a region of interest for cell number estimation. In each section, a contour was traced under a 5× objective around the LDT/SubLDT and around the PPT (Paxinos and Franklin, 2001; Fig. 1). Multi-channel image stacks (with 0.5-μm thickness for each optical section) were acquired under a 40× objective through the mounted histologic section. For the purpose of counting, a grid size of 250 × 150 μm^2^ and a counting frame of 120 × 120 μm^2^ were used. Within these images, all cells located below 1 μm from the surface through 15 μm of the section were counted. Counting was performed by moving through the z plane to assess the labeling for the EYFP on the plasma membrane of ACh and NonACh neurons in the LDT/SubLDT and PPT regions.

### Statistical analysis and representation of data

From the *in vivo* studies, electrophysiological and histochemical data were prepared and analyzed using MATLAB (R2011b, MathWorks) and SYSTAT (SYSTAT Software, version 13). Sections from most brains were analyzed for the estimated percentage of all ACh neurons which were EYFP positive. In addition, the proportion of units that were submitted to the juxtacellular labeling protocol (juxta-submitted) which were labeled for Nb (Nb-labeled) along with the proportion of these which were ACh positive or negative and EYFP positive or negative were computed per mouse. For the analysis of the recorded units, the electrophysiological data for all recorded and juxta-submitted units was computed. In offline analysis, units were classified as having responded or not responded by emitting a spike during the short (15 ms) light pulse and accordingly considered as pACh or pNonACh neurons. For assessment of whether these units corresponded to immunohistochemically identified Nb-labeled ACh or NonACh neurons, it was necessary to consider them as a group per mouse, since not all juxta-submitted units were Nb-labeled and not all Nb-labeled cells were ACh positive or negative in each brain. Accordingly, if on offline analysis, all juxta-submitted units in a brain were classified as pACh or pNonACh, and all Nb-labeled cells recovered in that brain were either ACh positive or negative, all such juxta-submitted units from that mouse were considered to represent and were thus collectively classified as Nb-labeled ACh positive or negative units, respectively. According to these criteria, none of the pACh units in the TG-A mice could be classified as Nb-labeled ACh units and are thus presented only as pACh units. For the TG-UA and WT-UA mice, on the other hand, the majority of juxta-submitted units could be collectively classified per mouse as Nb-labeled ACh or NonACh units. A small number of juxta-submitted units could also be collectively identified as Nb-labeled ACh or NonACh, EYFP positive or negative.

Measurements were compared statistically using Student’s t tests or in some cases one-way ANOVA repeated measures followed by *post hoc* within comparisons (using Fisher’s LSD, SYSTAT). Figures were prepared using MATLAB and Adobe Illustrator and Photoshop (Adobe Creative Suite, CS4).

## Results

### Photostimulation and identification of ACh neurons in ChAT-ChR2-EYFP TG mice

Neurons were recorded and photo-stimulated in ChAT-ChR2-EYFP TG mice within the LDT/SubLDT and PPT nuclei where ACh neurons are clustered in the PMT (Fig. 1). In a sample of TG mice (*n* = 12), the proportion of ACh PMT neurons (immunostained for either VAChT or ChAT), which appeared to express ChR2-EYFP (identified by intrinsic YFP or that enhanced by immunostaining for GFP), was assessed and estimated to be ∼83% (83.23 ± 3.06, mean ± SEM). Of all neurons with apparent ChR2-EYFP expression, ∼90% were ACh and ∼10% NonACh.

**Figure 2.**
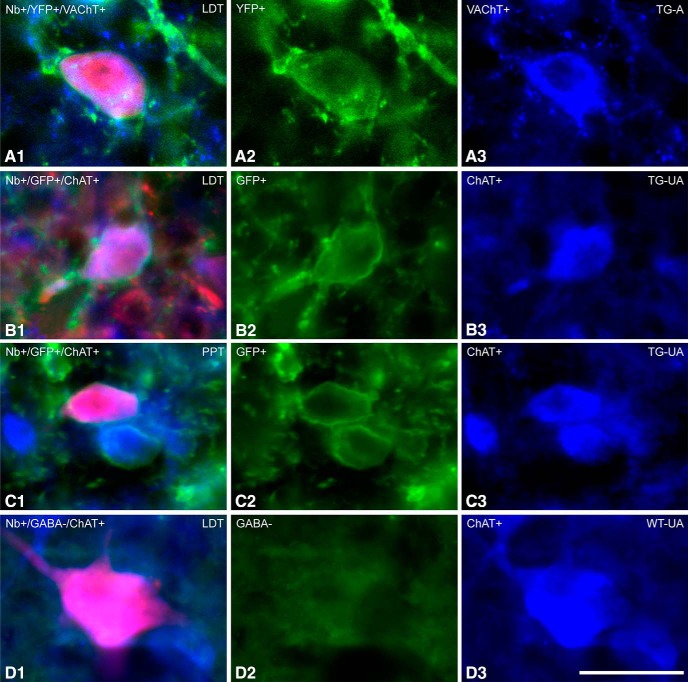
Fluorescent images of ACh recorded and Nb-labeled cells. Neurons which were recorded and labeled with Nb (in red) were identified as ACh neurons by immunofluorescent staining for VAChT or ChAT (in blue). ***A*,** Located in the LDT of a TG-A mouse (ChAT8), an Nb-labeled cell, which was VAChT+, appeared to express ChR2-EYFP (evident as YFP over the plasma membrane). ***B*,** Located in the LDT of a TG-UA mouse (Chronic (C)ChAT8), an Nb-labeled cell, which was ChAT+, appeared to express ChR2-EYFP (revealed by immunohistochemical staining for GFP over the plasma membrane). ***C*,** Located in the PPT of a TG-UA mouse (CChAT13), an Nb-labeled cell, which was ChAT+, also appeared to express ChR2-EYFP (revealed by immunohistochemical staining for GFP over the plasma membrane). ***D*,** Located in the LDT of a WT-UA mouse (CWT8), an Nb-labeled cell, which was ChAT+ was also confirmed to be immunonegative for GABA.

Putative cholinergic neurons were studied electrophysiologically in ChAT-ChR2-EYFP TG mice: *in vitro* within the LDT in brain slices and *in vivo* within the LDT/SubLDT and PPT in both A mice (*n* = 7) and UA, head-fixed, naturally sleeping-waking, mice (*n* = 8; Fig. 1). Based on previous *in vitro* studies (Ishibashi et al., 2015), neurons which produced photocurrents with photostimulation were considered to be pACh cells. Based on the *in vitro* results together with those from *in vivo* reports (Hedrick et al., 2016), units which emitted a spike during light pulses of ≤15 ms were also identified *in vivo* as pACh units. Of the units recorded *in vivo* in the LDT/SubLDT and PPT (*n* = 94 in 15 mice), approximately half were thus considered to be pACh units (*n* = 48) and the remainder, which did not respond, pNonACh units (*n* = 46). A number of the responding units (*n* = 42 or ∼62% of the total) and nonresponding units (*n* = 26 or ∼38% of the total) were submitted to juxtacellular labeling (juxta-submitted). Of the juxta-submitted units, ∼72% were successfully labeled with Nb (Nb-labeled, *n* = 50). Of the Nb-labeled cells, the majority were ACh positive (*n* = 34 or ∼68%) and the minority ACh negative (*n* = 16 or ∼32%). Of these neurons, approximately half were recovered in mice in which only responding or nonresponding units were juxta-submitted and in which all the Nb-labeled cells recovered were ACh (VAChT or ChAT immunopositive) or NonACh (VAChT or ChAT immunonegative) and were accordingly classified as representing Nb-labeled ACh or NonACh units (*n* = 20; see Materials and Methods). Of the responding Nb-labeled units, 90% were classified as ACh (*n* = 18; Fig. 2*A–C*) and 10% as NonACh (*n* = 2; data not shown).

From the *in vivo* recording studies, all Nb-labeled cells were also examined for expression of ChR2-EYFP by examination of the intrinsic YFP or that enhanced by immunostaining for GFP. In the LDT/SubLDT and PPT of both A and UA TG mice, the protein could be clearly visualized in the plasma membrane of only a proportion (∼56%) of all the Nb-labeled ACh positive neurons (Fig. 2*A–C*). Among responding Nb-labeled ACh units, however, a larger proportion (∼83%) were EYFP+. Nonetheless, since some (∼17%) of the responding Nb-labeled ACh units were also considered EYFP negative, it appears that the sensitivity for detection of the intrinsic YFP and the immunohistochemically enhanced YFP in the plasma membrane was not fully adequate for evaluation of positive versus negative expression of ChR2-EYFP in the Nb-labeled cells.

From the results of the *in vivo* studies, we concluded that the probability of the responding, “pACh units” being ACh neurons was ∼90%. For the group of “Nb-labeled ACh units,” the probability was 100% for the ∼72% of the juxta-submitted responding units, which were Nb-labeled, less 90% times the ∼28%, which were not Nb-labeled, and thus ∼97%.

### Electrophysiological study of ACh neurons in brain slices and A TG mice

**Figure 3.**
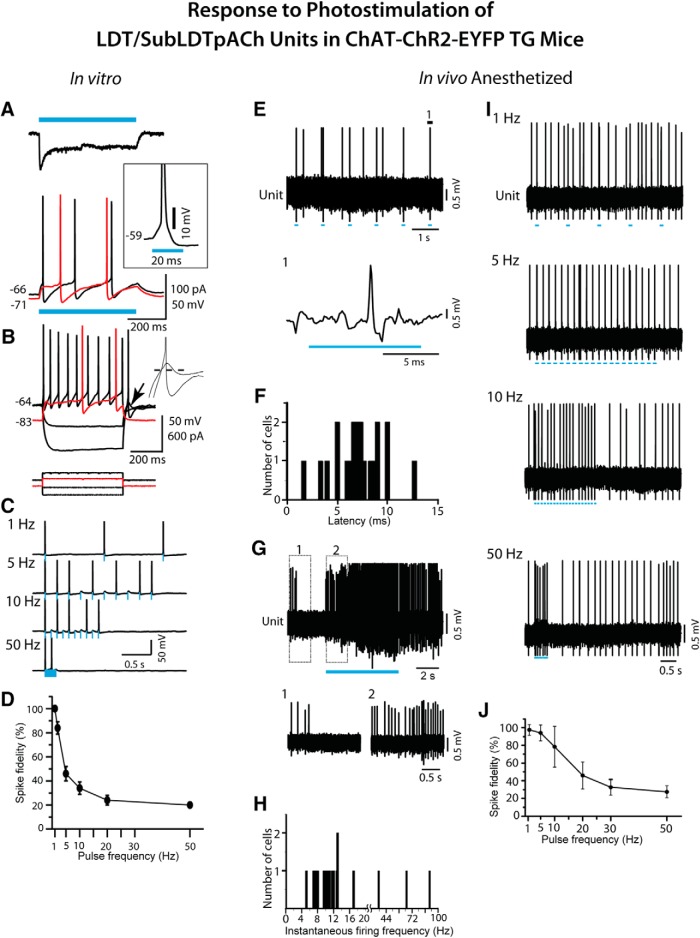
Response of LDT/SubLDT cholinergic units to blue light stimulation in ChAT-ChR2-EYFP TG mice. pACh units were studied *in vitro* (***A–D***) in brain slices and *in vivo* (***E–J***) in urethane-anesthetized mice. ***A***, *In vitro*, response of an LDT neuron to blue light stimulation (500-ms pulse, blue bars). Top trace illustrates the characteristic photo-current measured in voltage clamp mode. Inset illustrates the spike emitted during the light pulse at subthreshold potential (-59 mV) in current clamp mode. Bottom traces illustrate the depolarization and spiking produced by this photocurrent from two subthreshold membrane potentials (black trace, -66 mV; red trace, -71 mV). At -71 mV, the spike latency was greatly increased. ***B***, Membrane potential responses (top) of the same neuron to injected current pulses (bottom) from two membrane potentials (black traces -64 mV; red traces -83 mV). The intrinsic properties of this pACh neuron showed evidence of both A-current (delayed excitation from negative membrane potentials; red trace) and T-current (rebound excitation at termination of hyperpolarizing current pulses; arrow). Inset, Traces expanded around rebound. From -91 mV, the membrane potential rebound was subthreshold, but from -119 mV, it evoked a spike. Dashed line marks -64 mV. ***C***, Spiking produced by trains of 10 light pulses (10-ms duration) delivered at 1, 5, 10, and 50 Hz from ∼-60 mV for the same cell illustrated in ***A***, ***B***. Blue bars indicate the timing and duration of the light pulses. ***D***, Summary of spike fidelity of pACh LDT neurons versus light pulse frequency (mean ± SEM; *n* = 5). ***E***, *In vivo*, spiking in response to 10 light pulses (applied every second) by a pACh unit in a TG-A mouse. Segment of trace labeled 1 is expanded below (unit #12 in mouse ChAT6). ***F***, The spike latencies of units identified as pACh (*n* = 21). ***G***, During a long (∼5 s) continuous light pulse, pACh units fired repetitively at near maximal tonic discharge rates. Segments 1 and 2 are expanded below. ***H***, The instantaneous firing frequencies of pACh units (*n* = 18) during long (∼5 s) continuous light pulses. Note that the *x*-axis and its scale are split at 20 Hz to include higher frequencies. ***I***, Spiking of pACh unit (#12 in mouse ChAT6) in response to short (∼15 ms) light pulses at different frequencies. ***J***, Spike fidelity of pACh units to short pulse stimulation at different frequencies (mean ± SEM; *n* = 6).

The response to photostimulation of pACh neurons in the LDT was first studied *in vitro* in brain slices of ChAT-ChR2-EYFP TG mice (Fig. 3*A–D*). After patching and breakthrough in voltage clamp mode, light pulses (473 nm, 500 ms) were delivered to determine whether ChR2 photocurrents were present (Fig. 3*A*). When present (13/33 neurons), these currents were robust and showed a characteristic rapid initial transient (116 ± 24 pA, mean ± SEM) that developed with a time constant of ∼2 ms and then exponentially decayed (tau ∼20 ms) to a steady current (68 ± 13 pA; *n* = 5). After confirming functional expression of ChR2, recording was switched to current clamp, under which the photocurrents produced depolarizations (17.7 ± 1.2 mV; *n* = 5) that effectively evoked repetitive firing of three or four spikes during the 500-ms pulse (Fig. 3*A*, bottom traces). Spikes were reliably evoked with a latency measured from current onset to the foot of the action potential of 11.7 ± 1.5 ms (29 measurements from five neurons; Fig. 3*A*, inset), when tested from a subthreshold membrane potential (-59.8 ± 0.6 mV). Longer spike latencies (129–429 ms) occurred at more negative membrane potentials from which intrinsic currents appeared to delay the depolarization (-65 to -84 mV; Fig. 3*A*, bottom, red trace). Responses to direct current pulses (Fig. 3*B*) confirmed that the photoresponsive neurons had intrinsic membrane properties consistent with the presence of an A-type K^+^ current or of both an A-type K^+^ current and a T-type Ca^2+^ current, as previously described in guinea pig and rat ACh LDT neurons (Type II and III cells, respectively; Leonard and Llinas, 1990; Kamondi et al., 1992). Collectively, these data indicate that at membrane potentials around threshold, short duration light pulses should effectively drive firing of cholinergic PMT neurons in these mice. To test this in brain slices, the firing fidelity was measured using trains of different frequency light pulses (10-ms duration) from subthreshold membrane potentials near -60 mV (Fig. 3*C*,*D*). From these potentials, firing fidelity was high at 1–2 Hz but fell to 46% at 5 Hz and 20% at 50 Hz.

The response of neurons was subsequently studied in the LDT/SubLDT of urethane-anesthetized TG mice (52 units in seven mice; Figs. 1*A*, 3*E–J* ). All units were first tested with a short (∼10–15 ms) light pulse to determine their response. Of the units recorded, ∼40% responded within the short light pulse by emitting a spike during the light pulse and were thus considered to be pACh units (Fig. 3*E*). The units submitted to juxtacellular labeling included approximately half responding, pACh units (*n* = 18) and half nonresponding, pNonACh units (*n* = 19). Of neurons successfully labeled with Nb (*n* = 28), the majority were positively immunostained for VAChT (*n* = 17 or ∼61%) and the minority negatively so (*n* = 11 or 39%). As for the cell shown in Figure 2*A*, a proportion (35%) of the Nb+/VAChT+ cells also manifested intrinsic YFP in the plasma membrane confirming the expression of ChR2-EYFP in some of the Nb-labeled ACh neurons (Fig. 1*A*). However, in the TG-A mice, since none of the juxta-submitted units could be grouped as Nb-labeled ACh units, they were all simply classified as pACh or pNonACh according to their photo-response (see Materials and Methods).

The average latency to spike for the pACh units during the short pulse was 7.08 ± 0.54 ms (*n* = 21; Fig. 3*F*). Their average spike width during the pulse was 1.59 ± 0.01 ms (*n* = 17) and was not different from that during spontaneous spiking (1.60 ± 0.09 ms). With long (∼5 s) continuous light pulses, the pACh units fired continuously (Fig. 3*G*) at what appeared to be maximum average discharge rates (14.89 ± 3.72 Hz, *n* = 21) and most commonly with regular tonic spiking, although some units displayed higher frequency phasic spiking (*n* = 3; Fig. 3*H*), yielding an overall mean instantaneous firing frequency of ∼19 Hz across units (19.37 ± 5.40, *n* = 18). The pACh units were able to follow repetitive short light pulses fairly faithfully up to 5-10 Hz, above which their fidelity rapidly decreased to below 50% (Fig. 3*I*,*J*).

**Figure 4.**
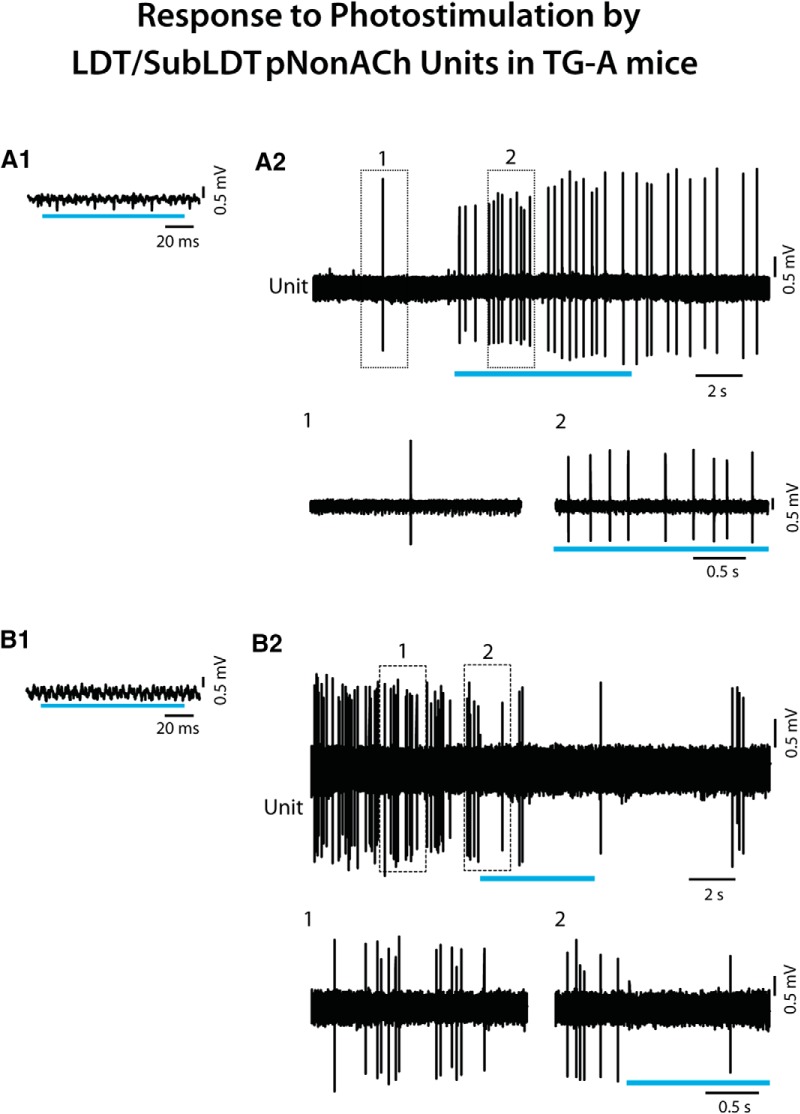
Response of LDT/SubLDT pNonACh units to long light pulses in TG-A mice. ***A1***, A pNonACh unit was identified by the failure to spike during short light pulses (here ∼100 ms; unit #3 in mouse ChAT6). ***A2***, Nevertheless, a long (∼5 s) continuous light pulse produced an increase in discharge, as illustrated in expanded segments (1, 2 below). ***B1***, Another pNonACh unit (#8 in mouse ChAT48) was identified by the failure to spike during short light pulses (here ∼100 ms). ***B2***, In this unit, firing decreased during a long (∼5 s) continuous light pulse, as illustrated in expanded segments (1, 2 below).

Although 60% of units recorded in the LDT/SubLDT did not respond within the short (∼15 ms) light pulse (*n* = 31) and were thus pNonACh units, almost all of these (∼97%) changed their average discharge rate during a long (∼5 s) continuous light pulse by either increasing (90%) or decreasing their rate (Fig. 4). Being highly variable, the mean latency to spike during the continuous light pulse for the pNonACh units, which increased their discharge, was 550.08 ± 217.87 ms (*n* = 12). It was nonetheless, shorter than the shift in EEG activity for these same units (820.83 ± 231.86 ms).

**Figure 5.**
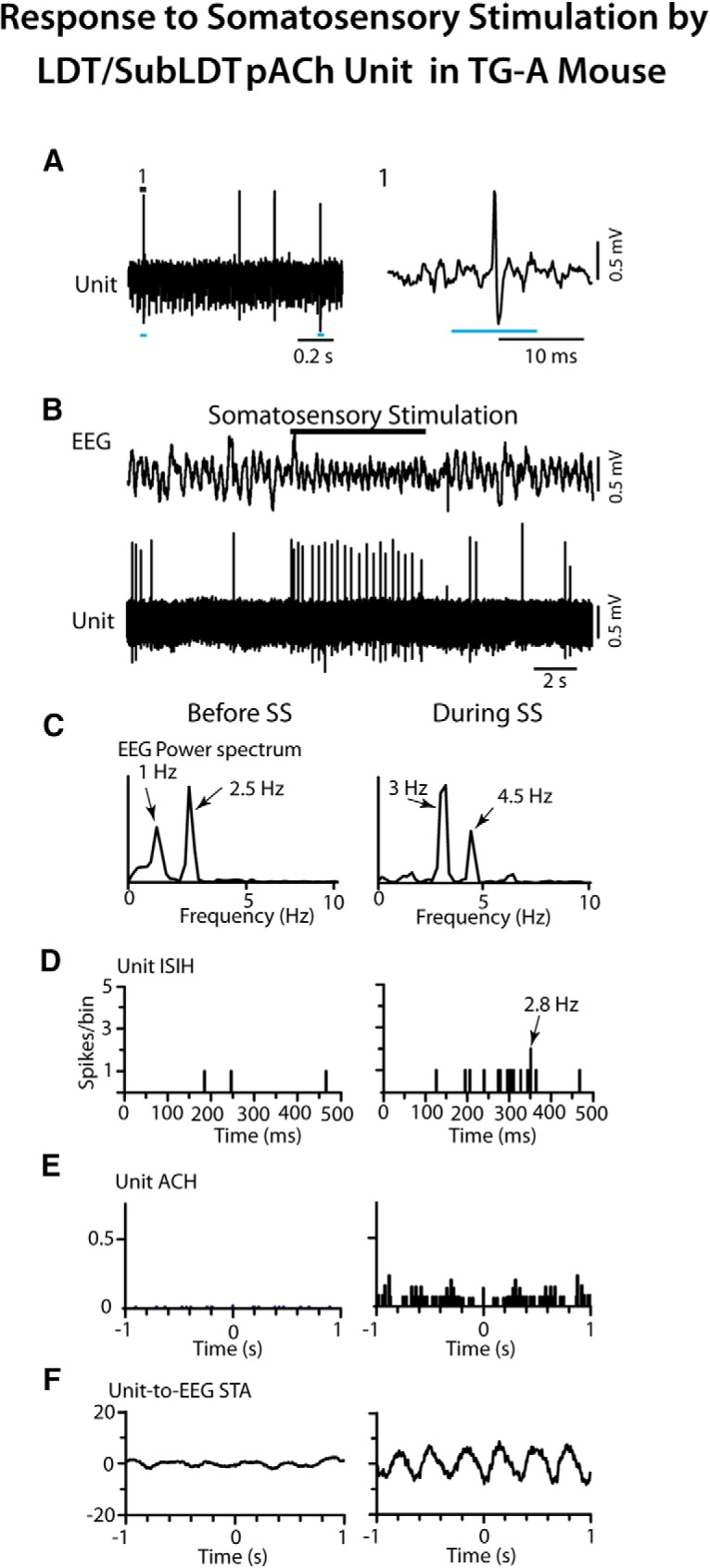
Response of LDT/SubLDT pACh unit to somatosensory stimulation (SS) in TG-A mouse. ***A***, Response of unit (#14 in mouse ChAT8) during the short (∼10 ms) light pulse stimulation (1, expanded on right) identifying it as a pACh unit. ***B***, The unit recorded in association with EEG activity recorded over retrosplenial cortex showed an increase in discharge rate during SS (tail pinch for ∼5 s) that preceded a shift in the EEG activity from irregular slow activity to RSA. ***C***, EEG power spectra showed the shift in peak frequencies during the SS into the range of the RSA (∼3–4.5 Hz). ***D***, The unit ISI histogram (ISIH) revealed instantaneous firing frequencies (as the reciprocal of the interval values with the mode indicated by arrow) around the frequency of the RSA, indicating tonic firing around this frequency during the stimulation. ***E***, In the unit autocorrelation histogram (ACH with correlation coefficients on vertical axes), the unit showed a certain degree of rhythmicity or regularity in its firing near the slow RSA frequency. ***F***, In the unit-to-EEG STAs (with mV on vertical axes), the unit spike train showed a degree of cross-correlation with the EEG RSA at ∼3 Hz. Before and during periods of analysis correspond to 5-s periods from EEG and unit activities illustrated above.

The response of the pACh units was subsequently examined in relation to the EEG to determine whether changes in unit response were associated with changes in EEG activity in the TG-A mice. First, it was established that changes in EEG activity recorded over the retrosplenial cortex could be induced in the urethane-anesthetized mice, as had previously been established and applied in urethane-anesthetized rats (Boucetta and Jones, 2009). Accordingly, it was found that somatosensory stimulation (tail pinch for ∼5 s) reliably elicited changes in the EEG typical of partial cortical activation as marked by a shift from irregular slow activity, similar to slow wave activity of SWS, to rhythmic slow activity (RSA), similar in pattern but of lower frequency to θ activity of active/attentive W (aW) or PS. Following identification of units responsive to the short light pulse, it was found that these pACh units responded to the (∼5 s) somatosensory stimulation by an increase in discharge that preceded the cortical activation (*n* = 3; Fig. 5*A*,*B*). As had been described for LDT/SubLDT ACh neurons in urethane-anesthetized rats (Boucetta and Jones, 2009), the firing of the pACh units in the urethane-anesthetized TG mice during somatosensory stimulation was tonic and here appeared to show a degree of rhythmicity or regularity in the spiking (at ∼3 Hz) that was somewhat cross-correlated with the slow θ EEG activity (at ∼3 Hz; Fig. 5*C–F*).

**Figure 6.**
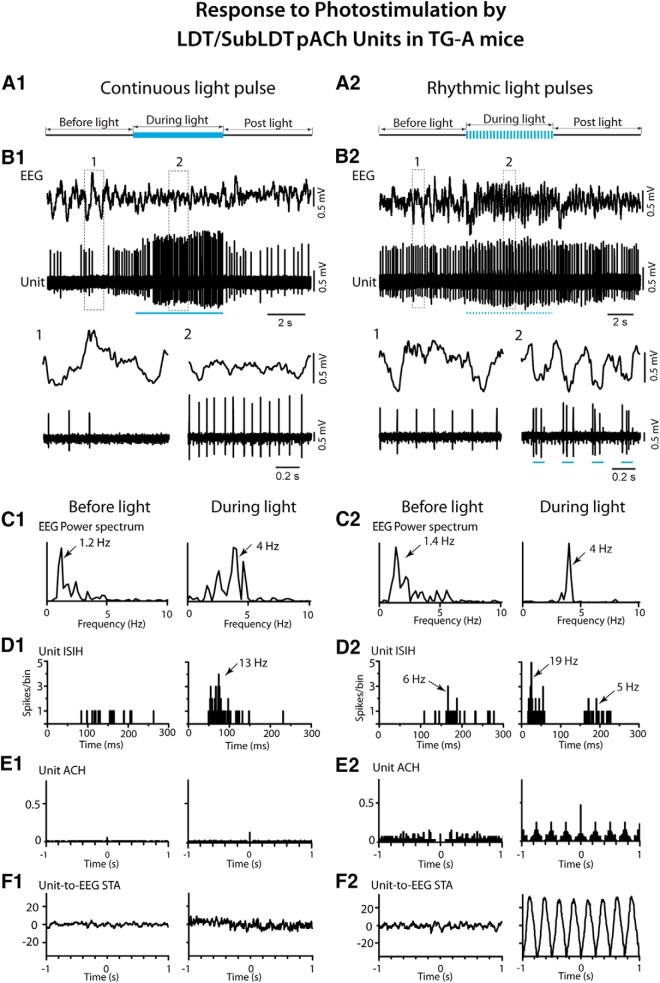
Response of LDT/SubLDT pACh units along with EEG to continuous and rhythmic light pulse stimulation in TG-A mice. ***A1***, A long (∼5 s) continuous light pulse was delivered during irregular slow activity. ***B1***, The pACh unit (#11 in mouse ChAT6) discharge and EEG activity changed during the light pulse, as shown in segments expanded from before (1) and during (2) photostimulation. The unit increased tonic firing, while the EEG shifted from irregular slow activity toward RSA. ***C1***, As evident in the power spectrum, EEG activity changed from irregular slow activity to RSA showing a peak at ∼4 Hz. ***D1***, As evident in the ISIH, the unit firing increased during the continuous light pulse with instantaneous firing frequencies up to ∼20 Hz and a single mode at ∼13 Hz, reflecting tonic firing. ***E1***, In the autocorrelation histogram (ACH), the unit showed no rhythmicity in its spiking. ***F1***, In the unit-to-EEG STA, the unit firing was not cross-correlated with the EEG RSA. ***A2***, A series of rhythmic light pulses (100 ms at 4 Hz for ∼5 s) were delivered during a period of EEG irregular slow activity. ***B2***, Changes in the discharge of the pACh unit (#13 in mouse ChAT6) and EEG activity were evident during the rhythmic light pulses as shown in the expanded traces (1, 2 below). The unit firing was driven by the pulses in a phasic manner in association with the EEG RSA. ***C2***, In the power spectra, the EEG shifted from irregular slow activity (∼1.4 Hz) before the rhythmic light pulses to RSA at 4 Hz during the rhythmic light pulses. ***D2***, In the ISIH, the unit firing manifested two modes during the rhythmic light pulses reflecting the phasic firing in clusters of spikes (with a mode 19 Hz) recurring at ∼4–6 Hz. ***E2***, In the ACH, the unit firing showed rhythmicity at ∼4 Hz. ***F2***, In the unit-to-EEG STA, unit activity was cross-correlated with the EEG RSA at 4 Hz. See legend to Figure 5 for graph details and abbreviations.

**Figure 7.**
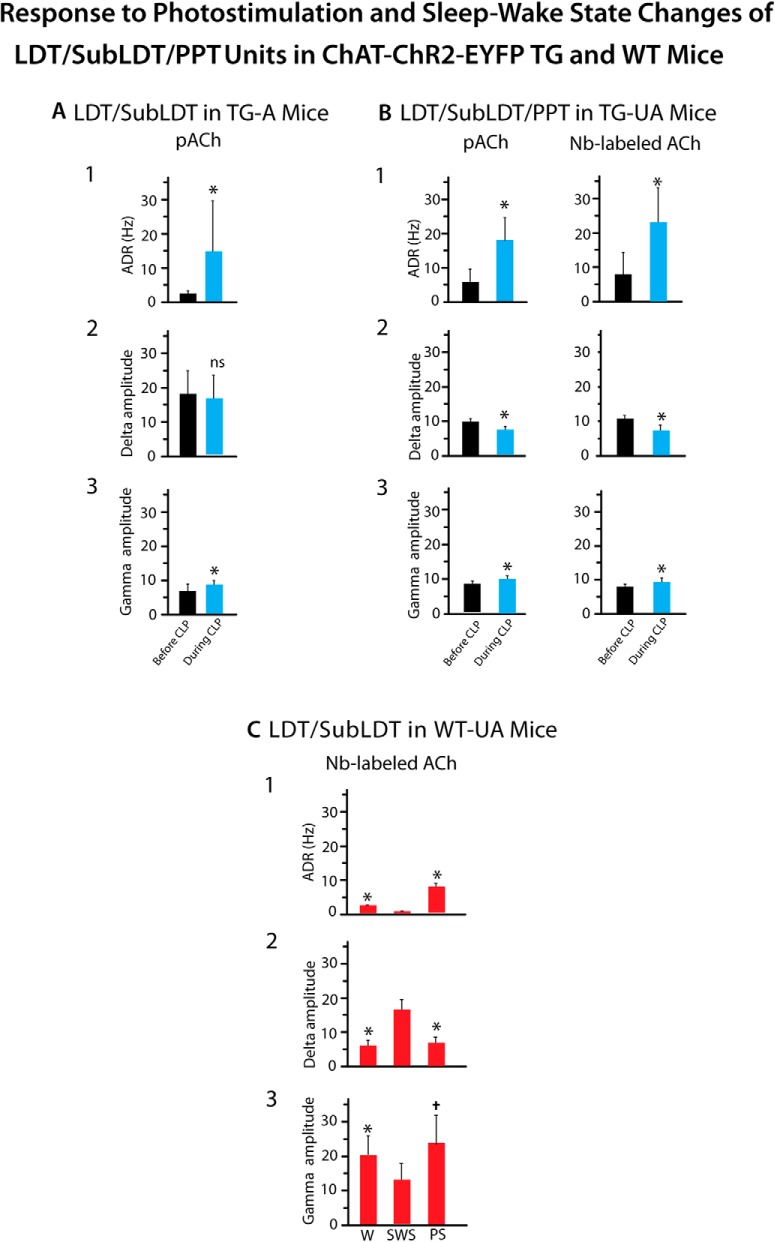
Response to photostimulation and sleep-wake state changes of LDT/SubLDT and PPT units in ChAT-ChR2-EYFP TG and WT mice. ***A***, ***B***, Effects of continuous light pulse (CLP) stimulation on average discharge rate (ADR), δ band EEG amplitude and γ band EEG amplitude. ***A***, In A (TG-A) mice, the average discharge rate of pACh units was significantly increased (1), the δ amplitude was unchanged (2), and γ amplitude was significantly increased (3) during the CLP, as compared to before the CLP (*n* = 18). ***B***, In UA (TG-UA) mice, the average discharge rate in all pACh units (left, *n* = 12) and Nb-labeled ACh units (right, *n* = 8) was significantly increased (1), δ amplitude significantly decreased (2), and γ amplitude was significantly increased (3) during the CLP as compared to before the CLP. * indicates difference between Before and During stimulation with *p* < 0.05 in ***A*** and ***B***. ***C***, In WT-UA mice, the discharge rate of Nb-labeled ACh units was significantly increased in W and PS as compared to SWS (1), the δ amplitude was decreased during W and PS as compared to SWS (2), and γ amplitude was significantly increased during W and PS as compared to SWS (3). * indicates difference between W and SWS with *p* < 0.05 and † indicates difference between SWS and PS with *p* < 0.05 in ***C***. See Results.

In response to a long (∼5 s) continuous light pulse, the pACh units increased their discharge before changes in EEG activity, which were marked by a shift from irregular slow activity to RSA over the retrosplenial cortex in the TG-A mice (Fig. 6*A1*,*B1*). The average latency to the first spike was 7.68 ± 0.713 ms and that estimated for the change in EEG activity 508.68 ± 101.74 ms (*n* = 19). The pACh units increased their average discharge rate from 2.56 ± 0.63 Hz before to 14.89 ± 3.72 during light stimulation (paired *t* = -3.65, df = 20, *p* = 0.002). The firing was most commonly tonic with a mean instantaneous firing frequency of ∼19 Hz across units and not cross-correlated with the RSA on the EEG during the light pulse (Fig. 6*C1–F1*). The change in EEG activity was marked by a significant increase in the mean peak frequency from 1.34 ± 0.20 to 3.65 ± 0.26 Hz (*n* = 19, paired *t* = -7.61, df = 18, *p* < 0.001) into the range of RSA. There was also an increase in mean amplitude of γ activity (30–58 Hz) from 6.34 ± 0.60 to 7.79 ± 5.20 mV (paired *t* = -2.45, df = 17, *p* = 0.025; Fig. 7*A*).

Delivery of rhythmic light pulses at a slow θ frequency (100 ms at 4 Hz for ∼5 s), similar to that evoked under urethane anesthesia, resulted in phasic spiking by the pACh units and a more marked shift from irregular slow activity to RSA in the EEG of the TG-A mice (Fig. 6*A2*,*B2* ). Their average discharge rate increased from 2.31 ± 0.60 Hz during pre-stimulation to 12.51 ± 3.97 Hz during stimulation (paired *t* = -2.64, df = 8, *p* = 0.030). With the train of rhythmic light pulses, the average latency to the first spike of the pACh units was 6.50 ± 1.03 ms during the first pulse, and that to the EEG change was 510.17 ± 122.35 ms (*n* = 12). The average latency to spike increased during successive pulses going from 6.11 ± 0.89 ms in the first pulse to 17.00 ± 1.38 ms in the second, 23.22 ± 4.64 ms in the third and 39.11 ± 9.84 ms in the fourth (with repeated measures ANOVA, *F* = 6.72, df = 3, 24; *p* = 0.002 and being significantly different between the first and all subsequent pulses by *post hoc* paired comparisons, *p* < 0.05). The units generally did not spike between pulses, and some units failed to spike at all during the successive 100-ms pulses. Although many units fired tonically (*n* = 5), some units fired in phasic clusters (21–79 Hz) or high-frequency bursts (≥80 Hz) of spikes during the rhythmic light pulses (*n* = 4), yielding an overall mean first mode of the instantaneous firing frequencies of 29.82 ± 9.08 Hz (*n* = 9). The bursts consisted most commonly of two spikes yet also up to three spikes. The mean of an intermediate mode was 9.1 ± 3.86 Hz, and that of a third mode was 4.22 ± 0.33 Hz (*n* = 9), reflecting the rhythmic phasic firing with the slow θ stimulation. The EEG was driven to a peak frequency of 4 Hz, at which frequency the unit phasic discharge was rhythmic and cross-correlated with EEG activity (*n* = 10; Fig. 6*C2–F2* ). These changes were associated with an increase in mean EEG γ activity from 6.77 ± 0.47 to 10.15 ± 0.72 mV (*n* = 9, paired *t* = -6.00, df = 8, *p* < 0.001).

### Study of ACh neurons in UA TG mice

The response and properties of neurons in the LDT/SubLDT and PPT were subsequently studied in head-fixed UA TG mice (*n* = 42 cells in eight mice; Fig. 1*B*,*D*). In these TG-UA mice, the majority of units selected for juxtacellular labeling were those which responded during the short light pulse and were thus considered pACh neurons (*n* = 27 or ∼77%) and a minority pNonACh neurons (*n* = 15 or ∼23%). Of the responding and nonresponding units, 71% were successfully labeled with Nb in the LDT/SubLDT (*n* = 14) and the PPT (*n* = 8). Of these Nb-labeled cells, the majority (*n* = 17 or ∼77%) were Nb+/ChAT+ in the LDT/SubLDT (*n* = 9) and PPT (*n* = 8), and of these, a minority (∼41%) were Nb+/ChAT+/EYFP+ in the LDT/SubLDT (*n* = 2) and PPT (*n* = 5; Figs. 1*B*,*D*, 2*B*,*C*). Of the juxta-submitted responding, pACh units, 18 units were classified as Nb-labeled ACh (above, with 6 in LDT/SubLDT and 12 in PPT), and of these, some were also positively immunostained for GFP over the plasma membrane (Fig. 2*B*,*C*), confirming positive expression of the ChR2-EYFP. Of juxta-submitted nonresponding, pNonACh units, two could be identified as Nb-labeled ChAT-negative and thus proven Nb-labeled NonACh neurons in the LDT/SubLDT (*n* = 2; data not shown). These Nb-labeled NonACh neurons were EYFP-negative.

**Figure 8.**
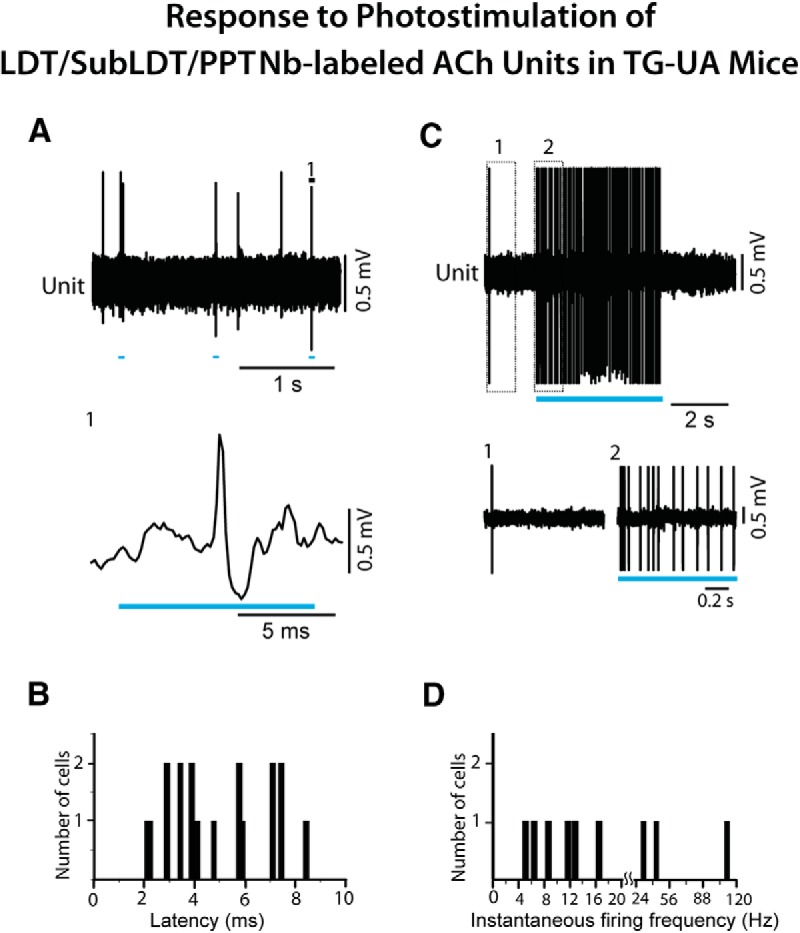
Response of LDT/SubLDT and PPT Nb-labeled ACh units to light stimulation in ChAT-ChR2-EYFP TG-UA mice. ***A***, Responses of a pACh unit (#4 in mouse CChAT13) to short (10 ms) light pulses (expanded segment 1, below). ***B***, The spike latencies for pACh units which were collectively identified as Nb-labeled ACh units (*n* = 18). ***C***, During a long (∼5 s) continuous light pulse, the pACh unit (#4 in mouse CChAT13) discharged at near maximal tonic discharge rates, as shown in expanded traces (1, 2 below). ***D***, The instantaneous firing frequencies of Nb-labeled ACh units (*n* = 9) during long (∼5 s) continuous light pulses. Note that the *x*-axis and its scale are split at 20 Hz to include higher frequencies.

Nb-labeled ACh units in the LDT/SubLDT and PPT of TG-UA mice showed short spike latencies during the (10–15 ms) short light pulse (Fig. 8*A*,*B*) with a mean latency of 4.89 ± 0.46 ms (*n* = 18), which did not differ significantly between the LDT/SubLDT and PPT cell groups. The mean latency for all pACh units in the TG-UA mice (5.09 ± 0.48 ms, *n* = 27) also did not differ from the Nb-labeled ACh units, but this mean did differ from that of the pACh units in the TG-A mice (*t* = 2.74, df = 46, *p* = 0.009), indicating that the latency was shorter in the UA mice. The average spike width of the Nb-labeled ACh units of the LDT/SubLDT and PPT in the TG-UA mice was 1.56 ± 0.10 ms during the light pulse and did not differ from that during spontaneous activity (1.52 ± 0.10 ms, *n* = 18). With a continuous long (∼5 s) light pulse, the Nb-labeled ACh units fired fairly continuously (Fig. 8*C*) at relatively high average discharge rates (23.02 ± 10.24 Hz, *n* = 9). Their spiking was most commonly regular tonic spiking with an average instantaneous firing frequency of ∼27 Hz (27.42 ± 11.27 Hz, *n* = 9); however, high-frequency phasic spiking was observed in a few units (*n* = 3; Fig. 8*D*). In none of the measures examined, were there systematic or significant differences between LDT/SubLDT and PPT cell groups, which are thus presented as one collective group.

**Figure 9.**
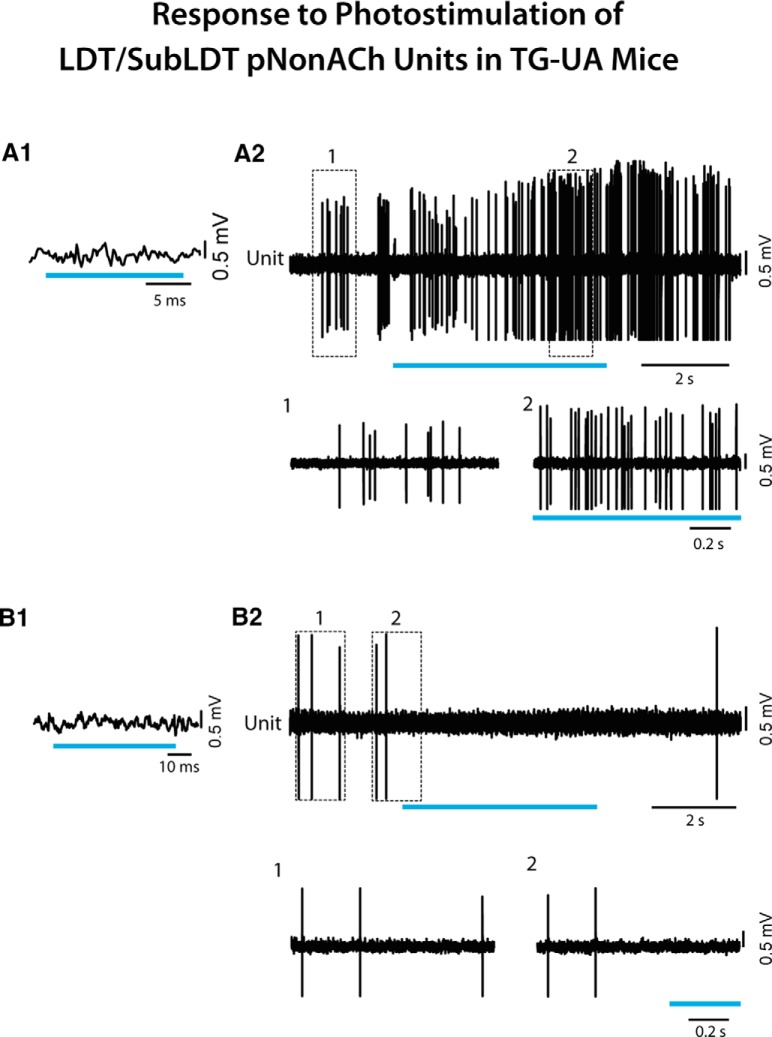
Response of LDT/SubLDT pNonACh units to continuous light pulse stimulation in TG-UA mice. ***A1***, A relatively short (∼15 ms) light pulse failed to evoke a spike in the unit (#2 in mouse CChAT7), which was thus considered pNonACh. ***A2***, A long (∼5 s) continuous light pulse resulted in increased discharge by the unit during the stimulation, as shown in expanded segments (1, 2 below). ***B1***, A relatively short (∼50 ms) light pulse failed to evoke a spike in the unit (#9 in mouse CChAT9), which was thus considered pNonACh. ***B2***, A long (∼5 s) continuous light pulse was marked by lack of discharge by the unit during the stimulation.

All the recorded units, which did not respond during a short (∼15 ms) light pulse and were thus considered pNonACh units in the LDT/SubLDT and PPT (*n* = 13), nonetheless changed their discharge rate during a long (∼5–20 s) continuous light pulse. The vast majority (77%) increased, whereas the minority decreased their rate of discharge (Fig. 9). One of those increasing its rate could be identified as Nb+/ChAT-negative, thus an Nb-labeled NonACh unit, which was also negative for GFP immunostaining and thus showing no evidence of ChR2-EYFP expression (data not shown). The average latency to spike during the long light pulse for the pNonACh units, which increased their rate, was 213.44 ± 98.80 ms (*n* = 9). The average latency to the shift in EEG activity associated with these same units was 305.33 ± 326.63 ms.

**Figure 10.**
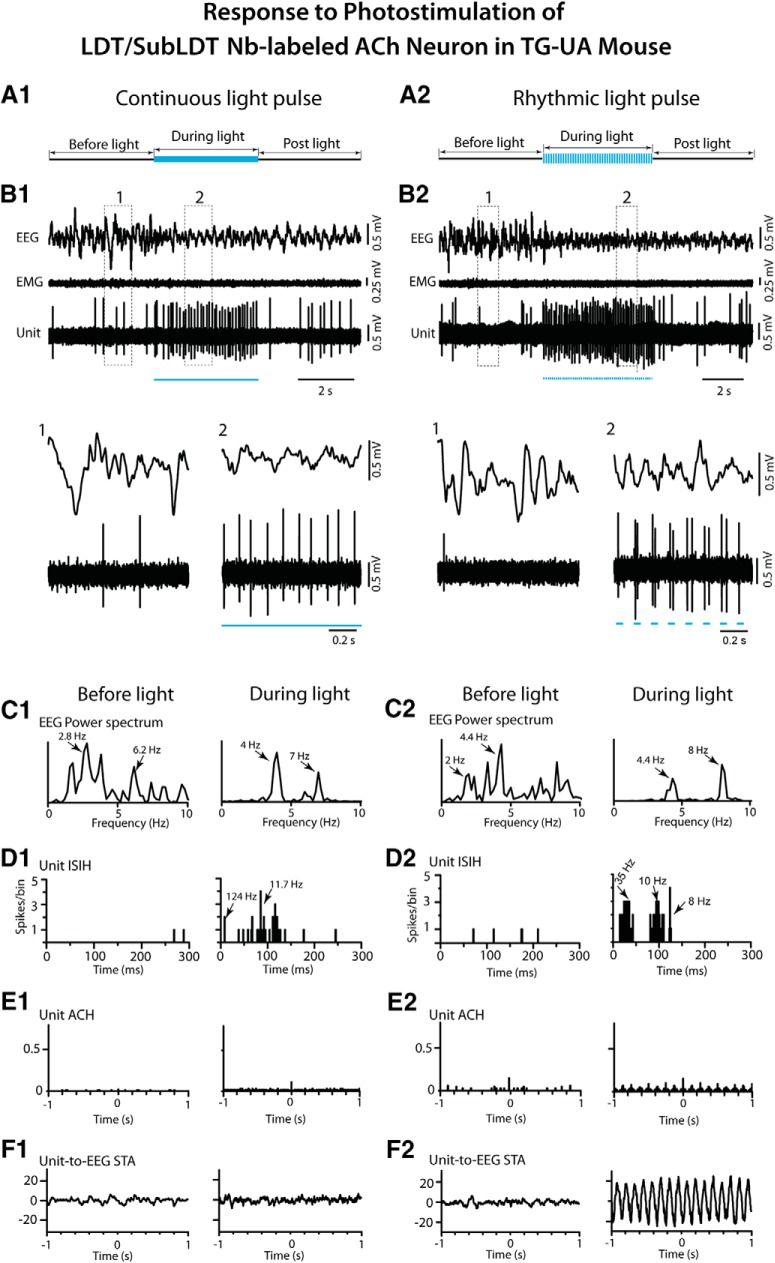
Response of LDT/SubLDT Nb-labeled ACh unit to continuous and rhythmic light pulse stimulation in a TG-UA mouse. ***A1***, A long (∼5 s) light pulse was delivered during a period of EEG irregular slow wave activity. ***B1***, The unit (#4 in mouse CChAT13) markedly increased its discharge while the EEG concomitantly shifted from irregular slow wave activity to more rhythmic θ activity, as shown in expanded segments (1, 2 below). ***C1***, In the EEG power spectra, the irregular slow wave activity with a peak at 2.8 Hz shifted to more regular mixed low and high θ activity with peaks at 4 and 7 Hz. ***D1***, The Unit ISIH showed increased activity reflecting mainly irregular tonic firing at ∼8–12 Hz (mode at 11.7 Hz indicated by arrow) with a few bursts (124 Hz indicated by arrow). ***E1***, In the unit autocorrelation histogram (ACH), there was no evidence of rhythmicity or regularity in the spike train. ***F1***, In the unit-to-EEG STA, there was no evidence of cross-correlation between the unit firing and EEG θ activity during the stimulation. ***A2***, Rhythmic light pulse stimulation at high θ frequency (50 ms, 8 Hz for ∼5 s) was delivered during a period of EEG irregular slow wave activity. ***B2***, The unit (#4 in mouse CChAT13) markedly increased its discharge while the EEG concomitantly shifted to rhythmic θ oscillation. The unit also fired phasically in association with the θ EEG activity, as illustrated in the expanded segments (1, 2 below). ***C2***, In the EEG power spectra, a shift was evident from mixed irregular slow activity before the light to rhythmic θ activity during the light with peaks at 4.4 and 8 Hz. ***D2,*** In the unit ISIH, three groups of ISIs reflected the phasic discharge in clusters of spikes (∼35-Hz mode indicated by arrow) along with a tonic rate (10-Hz mode, arrow) and recurrent θ rhythm (8-Hz mode, arrow). ***E2***, In the unit ACH, rhythmicity was evident at 8 Hz. ***F2***, In the unit-to-EEG STA, a cross-correlation of the unit activity with the EEG θ activity was evident at 8 Hz. See legend to Figure 5 for graph details and abbreviations.

**Figure 11.**
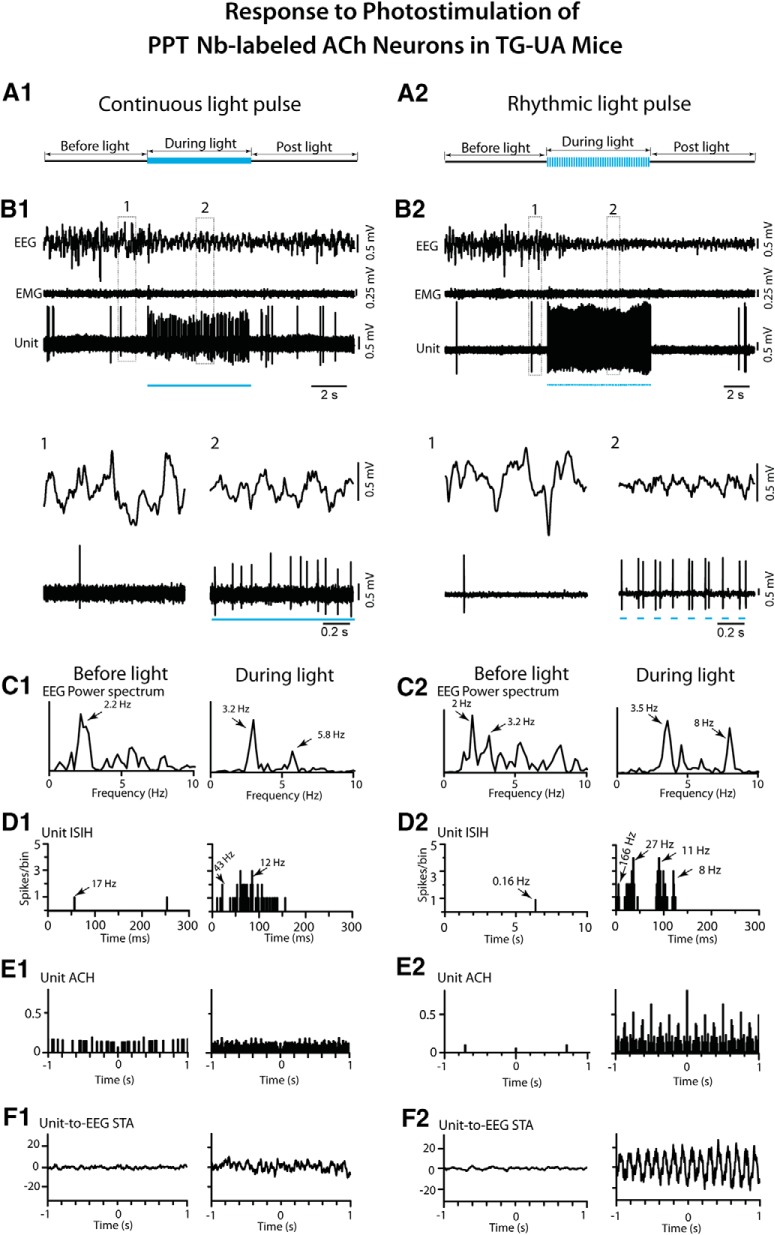
Response of PPT Nb-labeled ACh units to continuous and rhythmic light pulse stimulation in TG-UA mice. ***A1***, A long (∼5 s) continuous light pulse was delivered during a period of EEG irregular slow wave activity. ***B1***, The unit (#14 in mouse CChAT13) markedly increased its discharge while the EEG concomitantly shifted from irregular slow wave activity to more rhythmic θ activity, as shown in expanded segments (1, 2 below). ***C1***, In the EEG power spectra, a shift in peak frequencies from 2.2 to 3.2 and 5.8 Hz was apparent. ***D1***, In the unit ISIH, the increased discharge by the unit appeared to consist of irregular tonic firing (mode of 12 Hz indicated by arrow) with some phasic activity (∼43 Hz, arrow). ***E1***, In the unit ACH, there was no evidence of rhythmic firing. ***F1***, In the unit-to-EEG STA, there was no evidence of cross-correlated activity. ***A2***, Rhythmic light pulse stimulation at high θ frequency (50 ms, 8 Hz for ∼5 s) was delivered during a period of EEG irregular slow wave activity. ***B2***, The unit (#5 in mouse CChAT11) was driven to fire in a phasic manner with bursts of spikes with each light pulse, and the EEG was concomitantly driven to rhythmic θ oscillations, as shown in expanded traces (1, 2 below). ***C2***, In the EEG power spectra, the activity appeared to shift from mixed irregular slow wave activity to more rhythmic activity with peaks at 3.5 and 8 Hz. ***D2***, In the unit ISIH, the firing appeared to consist of phasic activity of clusters (with mode at 27 Hz, indicated by arrow) and some bursts (166 Hz, arrow) of spikes and tonic firing (mode at 11 Hz, arrow) along with the recurrent θ rhythm (mode at 8 Hz, arrow). ***E2***, In the unit ACH, a degree of rhythmicity in firing at around 8 Hz was apparent. ***F2***, In the unit-to**-**EEG STA, evidence of cross-correlated discharge of the unit with EEG θ activity 8 Hz was apparent. See legend to Figure 5 for graph details and abbreviations.

To examine the response of cholinergic LDT/SubLDT and PPT neurons in relation to changes in EEG activity in the TG-UA mice, a long (∼5 s) continuous light pulse was delivered when the EEG manifested irregular slow wave activity typical of SWS or the transition to SWS (tSWS; Del Cid-Pellitero et al., 2017; Figs. 10, 11). The Nb-labeled ACh units, along with pACh units, in the LDT/SubLDT and PPT, increased their rate of discharge during the continuous light pulse, while the EEG shifted from irregular slow wave activity to rhythmic low θ activity on which higher frequency waves were riding (Figs. 10, 11*A1*,*B1* ). There was no apparent change in EMG activity. For the Nb-labeled ACh units, the latency to spike during the long continuous light pulse was 4.67 ± 0.88 ms and that estimated for the shift in EEG activity was 533.11 ± 292.93 ms (*n* = 9). There was a significant increase in average discharge rate from a mean of 7.89 ± 6.50 to 23.02 ± 10.24 Hz (paired *t* = -3.30, df = 8, *p* = 0.011). The mean instantaneous firing frequency during the long light pulse was ∼27 Hz (see above), reflecting the most common tonic pattern of firing, despite phasic firing in a few units, which was not observed during spontaneous firing for which the mean instantaneous firing frequency was 14.80 ± 5.94 Hz (*n* = 9). The changes in unit firing were associated with an increase in the mean EEG primary peak frequency from 3.16 ± 0.32 to 4.32 ± 0.55 Hz, although not significant (paired *t* = -1.77, df = 8, *p* = 0.115) and in a secondary peak frequency from 3.57 ± 0.27 to 5.99 ± 0.50 Hz (paired *t* = -3.11, df = 4, *p* = 0.036), reflecting the emergence of a low θ rhythm. The unit discharge was not rhythmic or cross-correlated with the EEG during the continuous light pulse (Figs. 10, 11*C1–F1*). There was an associated decrease in the mean amplitude of δ activity (0.5–3.5 Hz) from 10.67 ± 1.04 to 7.30 ± 1.54 mV (paired *t* = 3.08, df = 7, *p* = 0.018) and increase in γ band activity (30–58 Hz) from 7.89 ± 0.812 to 9.50 ± 1.09 mV (paired *t* = -3.18, df = 7, *p* = 0.015), as was the case for all pACh units (Fig. 7*B*). These EEG changes were not observed in control WT-UA mice in which neither δ activity (going from 8.61 ± 3.24 to 8.66 ± 3.26 mV) nor γ activity (going from 4.65 ± 0.84 to 4.90 ± 0.92 mV; *n* = 3) were significantly different with presentation of long continuous light pulses. In the TG-UA mice, there was no change in EMG activity (30–100 Hz) going from 0.41 ± 0.09 to 0.40 ± 0.09 mV (*n* = 6) from the SWS period preceding to that period during the continuous light pulse. It thus appeared that the changes in ACh unit firing were associated with changes in EEG activity indicative of increased cortical activation but were not associated with behavioral arousal.

The effects of delivering rhythmic light pulses at a high θ frequency (50 ms at 8 Hz for ∼5 s), similar to that of active/attentive W or PS, was subsequently examined on the activity of the Nb-labeled LDT/SubLDT and PPT ACh units and the concomitant EEG activity in the head-fixed TG-UA mice (Figs. 10, 11). As seen with the long continuous light pulse, there was an increase in unit discharge and coincident shift in EEG activity with no change in EMG activity (Figs. 10, 11*A2*,*B2*). However, with the rhythmic light pulses, all Nb-labeled ACh units fired phasically, and EEG activity clearly shifted from irregular slow wave activity to rhythmic high θ activity. There was a significant increase in the mean average discharge rate from 2.28 ± 0.80 Hz before to 17.67 ± 2.81 Hz during light stimulation (paired *t* = -6.64, df = 13, *p* < 0.001). The mean latency to spike with the rhythmic light pulses was 4.00 ± 0.41 ms, and the latency to the shift in EEG activity was 185.06 ± 55.74 ms (*n* = 16). The spike latency increased during successive pulses going from 4.83 ± 1.06 ms during the first pulse to 10.83 ± 1.22 ms during the second to 11.67 ± 1.73 ms during the third to 16.83 ± 3.12 ms during the fourth pulse (in repeated measures ANOVA, *F* = 7.34, df = 3, 15, *p* = 0.011 with significant differences between the first and all subsequent pulses in *post hoc* comparisons, *p* < 0.05). The units did not spike between the pulses, and many units failed to spike within multiple 50-ms pulses that followed the first pulse. The mean instantaneous firing frequency of the first mode was at 113.77 ± 33.23 Hz, a second mode at 12.01 ± 1.88 Hz and a third mode at the θ frequency of the stimulation ∼8 Hz (8.13 ± 0.44 Hz; *n* = 14), indicating rhythmic phasic firing driven by the rhythmic light pulses at 8 Hz that was partially reflected in the EEG spectral changes (Figs. 10, 11*C2*,*D2*). With this phasic discharge, the Nb-labeled ACh units fired in spike clusters (21–79 Hz, *n* = 7) or high-frequency spike bursts (≥80 Hz, *n* = 7) during the light pulses. The bursts consisted most commonly of two spikes but also of three up to a maximum of five spikes. The mean primary EEG peak frequency increased from 3.09 ± 0.30 to 5.11 ± 0.49 Hz (paired *t* = -3.76, df = 15, *p* = 0.002) and a secondary peak from 3.40 ± 0.44 to 6.74 ± 0.51 Hz (paired *t* = -3.46, df = 12, *p* = 0.005). The Nb-labeled ACh units manifested rhythmic firing at 8 Hz which was cross-correlated with the high θ EEG activity at 8 Hz (*n* = 9; Figs. 10, 11*E2*,*F2*). There was an associated decrease in mean EEG δ band activity from 8.72 ± 0.60 to 5.14 ± 0.847 mV (paired *t* = 3.35, df = 14, *p* = 0.005) and an increase, though not significant, in γ band activity from 10.69 ± 0.47 to 11.63 ± 0.41 mV (paired *t* = -1.74, df = 14, *p* = 0.104). There was no change in nuchal EMG activity going from 0.41 ± 0.06 to 0.41 ± 0.06 mV. From video recordings, it could be seen that the vibrissae moved with the rhythmic light pulses, however the mice did not appear to awaken during the train of rhythmic light pulses. It thus seemed that the shift in EEG activity indicative of cortical activation was not accompanied by behavioral arousal during rhythmic light pulses.

**Figure 12.**
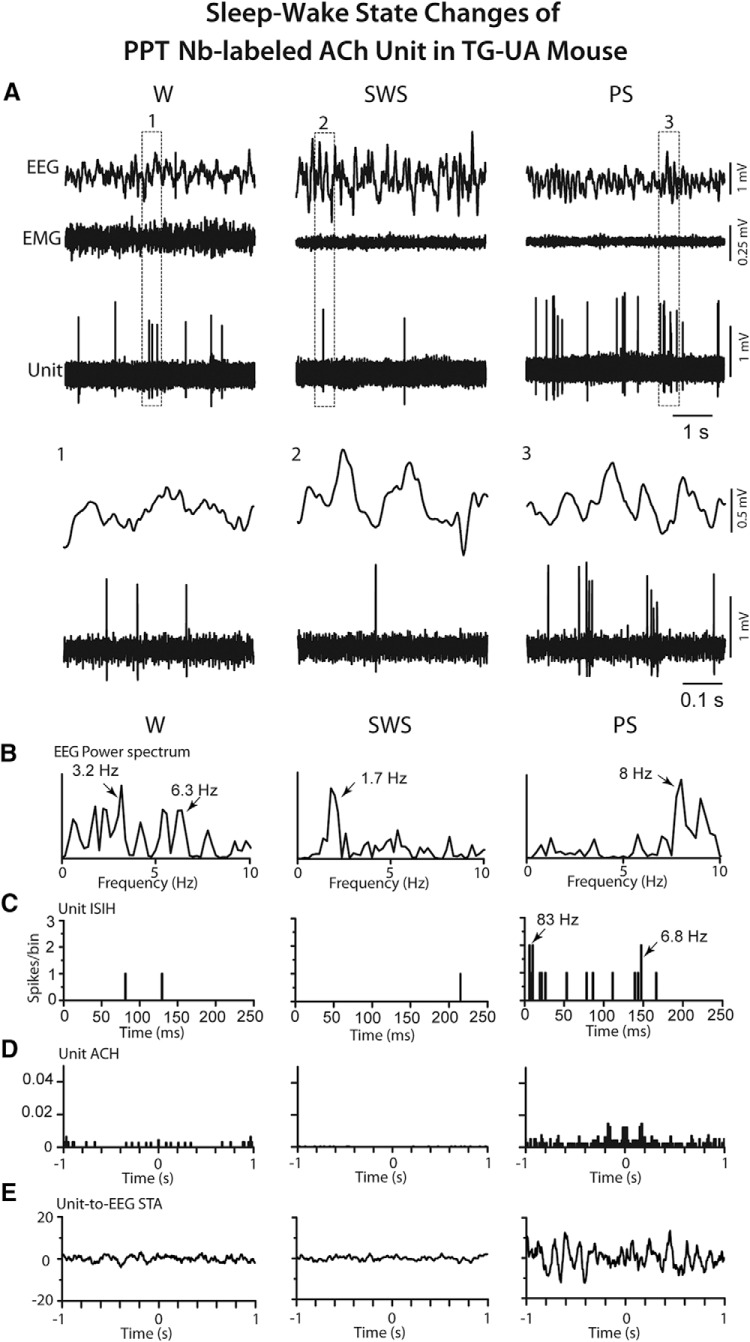
Response of PPT Nb-labeled ACh unit in TG-UA mouse to sleep-wake state changes. ***A***, The unit (#15 in mouse CChAT13) activity was recorded in association with EEG and EMG during W, SWS, and PS. The unit discharged during W and PS and was virtually silent during SWS. The pattern of firing also changed from mainly tonic during W to phasic with bursting during PS, as evident in expanded segments (1, 2, and 3 below) ***B***, In EEG power spectra, the activity was irregular and mixed during W, slow with a peak at 1.7 Hz during SWS, and rhythmic at θ frequencies of 8–9 Hz during PS. ***C***, In the unit ISIH, evidence of bursting activity (mode at 83 Hz indicated by arrow) is present but with minimal recurrent spiking (mode at 6.8 Hz, arrow) during PS. ***D***, In the unit ACH, there was little evidence of rhythmic firing. ***E***, In the unit-to-EEG STA, there was no evidence of cross-correlated rhythmic activity of the unit during high θ EEG activity of PS. Data analysis was performed on 5-s periods of W, SWS, and PS as illustrated. See legend to Figure 5 for other graph details and abbreviations.

To compare the discharge of cholinergic units and associated EEG activity during conditions of photostimulation with those during natural sleep-wake states, the discharge of pACh units along with EEG activity was successfully recorded in three pACh units which were also juxta-submitted in the head-fixed TG-UA mice. Of recovered Nb-labeled cells in these mice, two were in the LDT/SubLDT and one in the PPT (Fig. 12). The latter unit was classified as Nb-labeled ACh. As for this unit (Fig. 12*A*), all pACh units discharged at a lower average discharge rate during SWS (0.33 ± 0.13 Hz) as compared to W (14.97 ± 0.34 Hz) and PS (16.27 ± 8.68 Hz). The rate during SWS was much lower than that recorded before the light pulses during the photostimulation conditions, likely due to the higher amplitude slow wave activity during the undisturbed sleep in this sleep cycle condition. The rate during W and PS was similar to, although lower than, that recorded during continuous light pulses. The mean of the first mode of the instantaneous firing frequencies was high during W (42.33 ± 28.84 Hz) and very high during PS (123.10 ± 19.90 Hz; *n* = 3), reflecting high-frequency bursts of spikes (>80 Hz) in the pACh and Nb-labeled ACh units during PS (Fig. 12*B*,*C*). The bursts consisted of two or most commonly three to four spikes. This bursting activity during PS most resembled that seen during high θ rhythmic light pulse stimulation. However, in contrast to the latter (Fig. 11*E2*,*F2*), the bursting during PS was not rhythmic and was not cross-correlated with EEG high θ activity (Fig. 12*D*,*E*). The bursting occurred phasically and not continuously during PS. From the video recordings, it also appeared that whisker movements occurred phasically and not continuously during PS and often in association with bursting of the pACh units.

### Study of ACh neurons in naturally sleeping-waking WT mice

**Figure 13.**
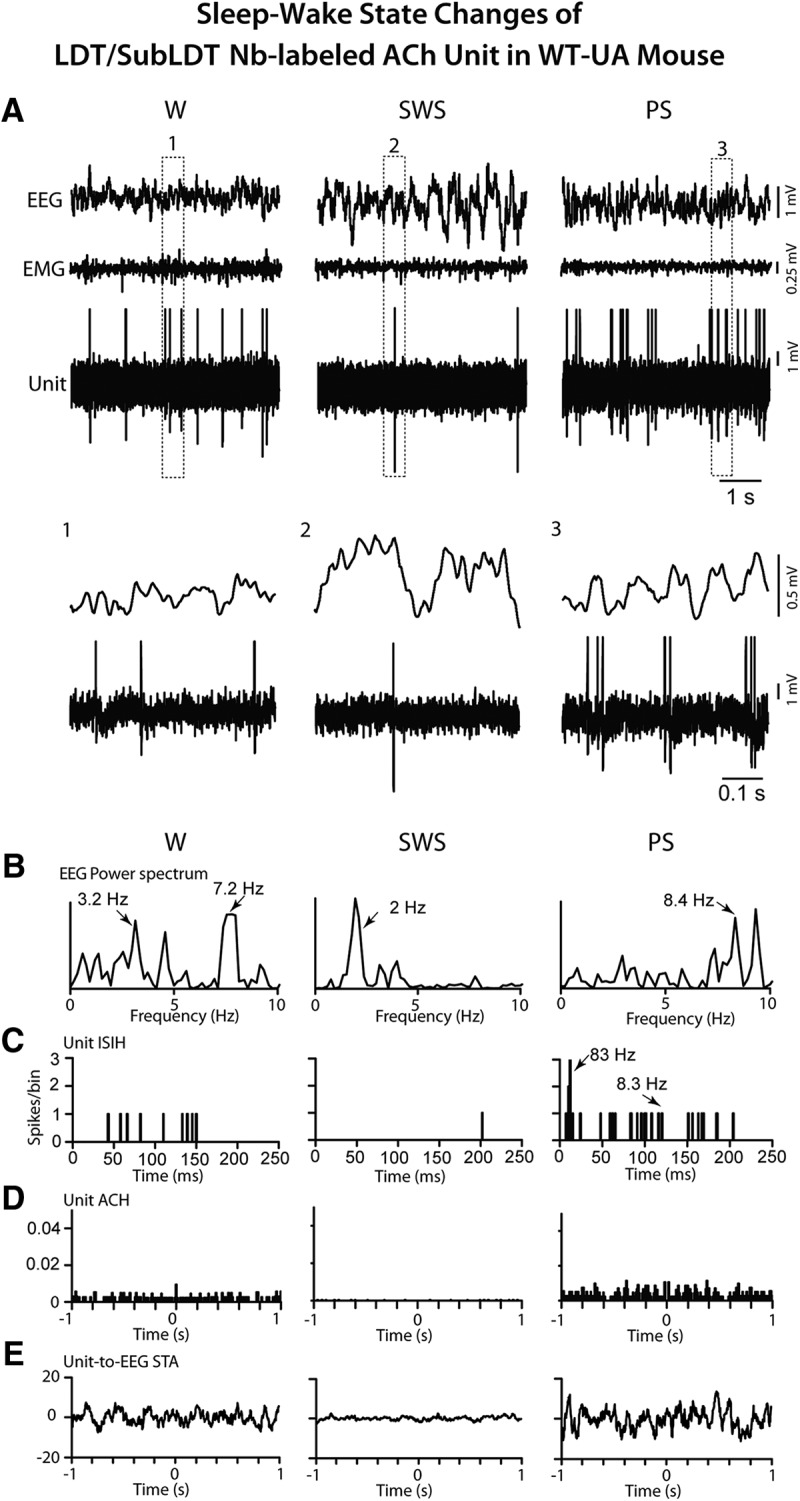
Response of LDT/SubLDT Nb-labeled ACh unit in WT-UA mouse to sleep-wake state changes. ***A***, The unit (#1 in mouse CWT8) activity was recorded in association with EEG and EMG during W, SWS, and PS. The unit discharged during W and PS and was virtually silent during SWS. The pattern of firing also changed from mainly tonic during W to phasic with bursting during PS, as evident in expanded segments (1, 2, and 3 below). ***B***, In EEG power spectra, the activity was irregular and mixed during W with a peak at 7.2 Hz (indicated by arrow), slow with a peak at 2 Hz during SWS and rhythmic at high θ frequencies of 8–9 Hz during PS. ***C***, In the unit ISIH, evidence of bursting activity (mode at 83 Hz indicated by arrow) is present but with little evidence of recurrent rhythmicity at the EEG peak frequency (8.3 Hz indicated by arrow) during PS. ***D***, In the unit ACH, there was no evidence of rhythmic firing. ***E***, In the unit-to-EEG STA, there was no evidence of cross-correlated rhythmic activity of the unit during high θ EEG activity. Data analysis was performed on 5-s periods of W, SWS, and PS as illustrated. See legend to Figure 5 for other graph details and abbreviations.

Neurons were recorded in the LDT/SubLDT of three UA head-fixed WT mice and submitted to juxtacellular labeling with Nb for subsequent identification as ACh neurons. Being selected according to the characteristics found in the Nb-labeled ACh units of the TG mice, four juxta-submitted units were successfully recorded across the three states of W, SWS, and PS that were among Nb-labeled neurons which were positively immunostained for ChAT (Fig. 2*D*). As evident in one Nb-labeled ACh unit (Fig. 13*A*), all the Nb-labeled ACh units were virtually silent during SWS, showed irregular tonic firing during W and phasic firing during PS. They all discharged at a lower rate during SWS (0.50 ± 0.058 Hz) than during W (2.38 ± 0.38 Hz) and PS (7.60 ± 4.36 Hz; with repeated measures ANOVA, *F* = 24.27, df = 2, 6; *p* = 0.016) and with significant differences between all states (with *post hoc* paired comparisons, *p* < 0.05). The different rates of ACh unit discharge were associated with different amplitudes of δ and γ activities across the three states (Fig. 7*C*). δ Activity was higher during SWS (16.13 ± 3.47 mV) than during W (5.73 ± 1.78 mV) and PS (6.39 ± 2.04 mV; with repeated measures ANOVA, *F* = 22.24, df = 2, 6; *p* = 0.015 with significant differences between SWS and W and SWS and PS, *p* < 0.05). γ Activity was lower during SWS (12.91 ± 5.01 mV) than during W (20.13 ± 5.93 mV) and PS (23.53 ± 8.39 mV; with repeated measures ANOVA, *F* = 5.15, df = 2, 6; *p* = 0.050 with significant differences between SWS and W, *p* < 0.05 and a trend between SWS and PS, *p* = 0.074). All units also displayed a different pattern of discharge going from commonly tonic firing during W to high-frequency spike bursts (>80 Hz) during PS, as reflected in the means of the first mode of the instantaneous firing frequencies during W (13.75 ± 1.47 Hz) and PS (101.83 ± 13.88 Hz; paired *t* = -5.35, df = 3, *p* = 0.013). The bursts consisted most commonly of two spikes but also of three to four spikes. The bursting during PS occurred in association with high θ EEG activity but was not rhythmic or cross-correlated with the EEG θ activity (Fig. 13*B–E*). It occurred phasically and not continuously during PS.

## Discussion

By application of optogenetics with juxtacellular recording and labeling, we discovered that ACh PMT neurons have the capacity to attenuate cortical irregular slow wave activity and promote rhythmic θ activity along with high-frequency γ activity in A and UA mice. The ACh neurons appear to be able to do so by tonic or phasic discharge which occurs naturally during W and PS.

### Role of ACh PMT neurons in cortical activity

Photostimulation of the ACh PMT neurons evoked decreases in irregular slow wave activity and increases in rhythmic θ activity together with increases in high-frequency γ recorded over the retrosplenial cortex in both A and UA mice. With a continuous light pulse that stimulated the ACh neurons to fire at near maximal rates in a tonic mode (10–20 Hz), the EEG shifted from irregular slow wave to rhythmic θ indicating that tonic firing by the ACh neurons both attenuates irregular slow wave activity and promotes rhythmic θ activity in limbic cortex. With rhythmic light pulses that drove the ACh neurons to fire rhythmically in bursts at 4 Hz in urethane-anesthetized or 8 Hz in UA mice, the EEG was driven in synchrony. The results suggest that the ACh PMT neurons can fire phasically and rhythmically, like ACh basal forebrain (BF) neurons (Lee et al., 2005) and thus possibly drive limbic θ. Indeed, the *in vitro* profile of the pACh neurons here showed membrane responses resulting from a A-type K^+^ and T-type Ca^2+^ currents, which were previously found in ACh PMT neurons (Leonard and Llinas, 1990; Kamondi et al., 1992) and ACh BF neurons (Khateb et al., 1995) and could contribute to rhythmic bursting by both cell groups. Consistent with the actions of A- and T-currents, the ACh PMT neurons here showed an increasing latency to spike and an increasing tendency to fire in bursts with successive rhythmic light pulses, which would be expected for rhythmic depolarization arising from presumed membrane hyperpolarization between pulses. However, such rhythmic phasic firing in synchrony with θ activity was not observed in the naturally sleeping-waking mice during either W or PS, as it also had not previously been found in rat ACh PMT neurons (Boucetta et al., 2014) although had in ACh BF neurons (Lee et al., 2005; Hassani et al., 2010). On the other hand, phasic firing in bursts was observed during PS here in the ACh PMT neurons, although it was neither rhythmic nor cross-correlated with θ, but irregular and associated with phasic behavioral activity of PS seen most prominently in rodents, as rapid whisker movements or twitches (Tiriac et al., 2012). We must conclude that the ACh PMT neurons can promote rhythmic θ activity by ACh release, which can occur with their tonic firing but likely in greater amounts with phasic bursting. Accordingly, the ACh PMT neurons can attenuate slow wave activity and facilitate both θ and γ activity, although do not appear to pace θ activity, as was recently also concluded for the cholinergic septo-hippocampal neurons (Vandecasteele et al., 2014). That ACh can promote rhythmic firing of target neurons through muscarinic receptors has been shown *in vitro* in hippocampus and limbic cortex (Konopacki et al., 1987; Dickson and Alonso, 1997).

Here, the photo-activation of the ACh PMT neurons would be expected to activate target neurons which are normally involved in stimulating cortical activation with θ and γ activity. Such target neurons can be in very local areas, including as shown here, within the LDT/SubLDT or PPT, where NonACh neurons were modulated by facilitation (>70%) or attenuation (<30%). In fact, the neurons in these nuclei are comprised by equivalent proportions of glutamate (Glu) and GABA together with the ACh neurons, each of which have different discharge profiles and play different roles in EEG activity and sleep-wake states (Boucetta et al., 2014). Approximately one half of the Glu and GABA neurons discharge like the ACh neurons maximally during W and PS (as W/PS-max active neurons) with cortical activation and could thus well be facilitated by the ACh neurons and participate in stimulating cortical activation. In addition, another large proportion of the Glu (∼30%) and GABA (∼45%) neurons discharge maximally during PS (as PS-max active) and could well also be facilitated by the ACh neurons. A remaining, minor proportion of the Glu neurons (∼20%) discharge maximally during W (as W-max active) with muscle tone and motor activity and could be among neurons whose discharge is attenuated by the ACh neurons. In addition, ACh fibers and terminals from the ACh PMT neurons provide a rich innervation to the oral pontine reticular formation (Satoh and Fibiger, 1986; Hallanger and Wainer, 1988; Jones, 1990; Semba et al., 1990), where carbachol can elicit θ activity through facilitating the discharge of the oral pontine reticular neurons (Vertes et al., 1993). Since the reticular neurons discharge tonically, however, they were thought to elicit θ activity through projections onto rhythmically discharging neurons located rostrally in the supramammillary and medial mammillary nuclei (Vertes and Kocsis, 1997), where ACh PMT neurons also project and carbachol also elicits limbic θ (Ariffin et al., 2010). ACh terminals are particularly rich within other cell groups within the pontine tegmentum (Jones, 1990), including most importantly the neurons in the ventral tegmental nucleus, which burst in phase with θ and project to the medial mammillary nuclei (Bassant and Poindessous-Jazat, 2001; Kocsis et al., 2001). The ACh PMT neurons could also act on more distant targets, including the anteroventral thalamic nucleus to which the ACh PMT neurons densely project (Satoh and Fibiger, 1986), as do the medial mammillary neurons, and where θ has been recorded and can be projected on the retrosplenial and limbic cortices (Dillingham et al., 2015). The promotion of θ by the ACh PMT neurons during W and during PS could serve, as is the case for septo-hippocampal neurons, an important role in memory consolidation during these states (Vertes and Kocsis, 1997; Boyce et al., 2016).

### Role of ACh neurons in the states of W and PS

With continuous light pulses and rhythmic light pulses (for 5 s) in the UA mice, the ACh neurons appeared to be able to stimulate cortical activation, which occurs naturally during W and PS. Indeed, in the naturally sleeping-waking states here, the ACh PMT neurons increased their discharge in association with high γ activity during both of these states relative to SWS, when these neurons were virtually silent, as similarly observed in rats (Boucetta et al., 2014). In response to the photostimulation applied during SWS, the increased tonic or rhythmic activation of the ACh neurons effectively shifted the EEG activity from one of SWS to one of cortical activation but without behavioral arousal of W during the stimulation. On the other hand, rhythmic stimulation of the ACh neurons evoked whisker movements, which occur naturally during W but also during PS (Tiriac et al., 2012). Yet, there was no change in neck muscle tone during the photostimulation to indicate either awakening with movement or PS with postural muscle atonia in the head-fixed mice. It is known from early pharmacological studies that local microinjection of carbachol or cholinesterase inhibitors into the oral pontine reticular formation can elicit cortical activation accompanied by muscle atonia and thus PS, which is dependent on muscarinic ACh receptors (Baghdoyan et al., 1984b; Gnadt and Pegram, 1986; Velazquez-Moctezuma et al., 1989; Reid et al., 1994). Yet, such microinjections have also elicited cortical activation with W (Deurveilher et al., 1997), calling into question the precise role of the ACh PMT neurons (Luppi et al., 2006). Similar ambiguity arises from optogenetic studies, since in a recent report, others claimed that photostimulation of the ACh LDT and PPT neurons in freely moving mice increased the probability of occurrence of PS (Van Dort et al., 2015). However, they stimulated the ACh neurons for prolonged periods (60, 80 and 120 s) and examined the probabilities of state change during long periods (30 s up to 3 min) following the photostimulation. In absence of recording, the discharge of the ACh neurons during these prolonged periods cannot be known. Moreover, unlike mice used in our study, the BAC TG mice used by Van Dort et al., contain ∼50 extra copies of the VAChT gene and display higher levels of VAChT mRNA, higher levels of VAChT protein, higher amounts of ACh release and a hypercholinergic behavioral phenotype (Kolisnyk et al., 2013; Crittenden et al., 2014), all of which calls into question the interpretation of findings derived from that model. Other investigators found that photostimulation of ACh neurons in the BF (for 15–20 s) stimulated transitions from SWS to a state of cortical activation with θ, which was most often associated with W but also, however, much less frequently with PS (Han et al., 2014; Irmak and de Lecea, 2014). Another study using chemogenetic activation of the ACh PPT neurons in freely moving animals reported attenuating EEG δ activity during natural SWS without promoting arousal or any change in the amounts of W, SWS, or PS (Kroeger et al., 2017), which would not be in contradiction with our optogenetic results, although only 20% of the ACh neurons apparently expressed the DREADD receptor. Most important however, the *in vivo* discharge of the ACh neurons during the chemogenetic stimulation was not known. In this same study, chemostimulation of Glu PPT neurons evoked W, complete with cortical activation and behavioral arousal with muscle tone. These effects could be due to the activation of the majority of the Glu neurons in this area, which were previously found to discharge maximally during W and PS, like the ACh neurons, and also of a small minority of the Glu neurons found to discharge maximally during W in positive correlation with EMG (Boucetta et al., 2014). However, the conclusion that PPT Glu neurons promote W ignores the finding that a third of the Glu neurons were previously found to discharge maximally during PS and accordingly elicits caution concerning interpretations of results based solely on opto or chemo stimulation in absence of electrophysiological recordings. We conclude with confidence from our optogenetic studies which combined photostimulation with recording of identified neurons that the ACh PMT neurons promote cortical activation of W and PS and not behavioral arousal of W.

### How do ACh PMT neurons promote cortical activation with W or PS?

The ACh PMT neurons were found to have the capacity to discharge high-frequency bursts of spikes driven by photostimulation but also present during naturally occurring PS. Yet, the bursts were not continuous, rhythmic or cross-correlated with limbic cortical θ activity during natural PS. The irregular bursts appeared to be associated with the phasic activity of PS seen in the mice as rapid whisker movements. A role for ACh PMT neurons in whisker movements has been proposed to occur through projections to whisker-related sensory-motor regions in forebrain and brainstem (Beak et al., 2010). Similar bursting neurons whose discharge was related to phasic activity in PS were previously recorded in the LDT of mice and proposed to be putative cholinergic neurons (Sakai, 2012). Indeed, neurons in the PPT which were first recorded in cats and proposed to be cholinergic, burst in association with phasic ponto-geniculo-occipital (PGO) spikes recorded in the lateral geniculate to which the ACh neurons project (Sakai and Jouvet, 1980). Release of ACh was shown to be significantly higher in the lateral geniculate during PS than during W and SWS (Kodama and Honda, 1996), which would likely be due to the burst discharge by the ACh PMT neurons during PS. Moreover, PGO spikes were shown to depend on activation of nicotinic receptors within the lateral geniculate (Hu et al., 1988), and neurotoxic lesions of the PMT neurons resulted in a loss of PGO spiking with PS, supporting a critical role for the ACh PMT neurons in this phasic activity of PS (Webster and Jones, 1988).

Being virtually silent during SWS, the ACh PMT neurons discharge during W and PS in association with cortical θ and γ activity, which they evoke when stimulated during SWS. From these collective results, we conclude that the ACh PMT neurons have the capacity to stimulate cortical activation with rhythmic θ and γ by their tonic or phasic discharge during W and PS, likely through actions of ACh on muscarinic receptors on target neurons. Through their burst discharge, they can further facilitate phasic activation of sensory-motor and activating systems in the brainstem and forebrain, likely through additional actions of ACh on nicotinic receptors and prominently so during PS. Although no evidence for a role in the tonic muscle atonia of PS was evident in the present study, this role of cholinergic neurons likely involves modulation of other brainstem circuits in part through different muscarinic receptors (Brischoux et al., 2008). But just as waking depends on the complex involvement of different arousal and sleep-wake networks, PS depends on specific orchestration of these systems, including the silencing of the aminergic and orexinergic arousal systems, which the activation of ACh PMT neurons cannot likely orchestrate alone or without the long preparatory period of SWS which normally precedes PS (Jones, 2017).

## Synthesis

Reviewing Editor: Rae Silver, Columbia University Decisions are customarily a result of the Reviewing Editor and the peer reviewers coming together and discussing their recommendations until a consensus is reached. When revisions are invited, a fact-based synthesis statement explaining their decision and outlining what is needed to prepare a revision will be listed below. The following reviewer(s) agreed to reveal their identity: NONE. Note: If this manuscript was transferred from JNeurosci and a decision was made to accept the manuscript without peer review, a brief statement to this effect will instead be what is listed below.

The authors have responded splendidly to the previous review. That said, the clarity of the rebuttal is not as clear in the revised text. Please revisit the material submitted, and revise the manuscript to sharpen the questions raised (Introduction) and Discussion of the contribution of the present work. The research itself is lovely.

Additionally, a visual abstract will help the reader.

## Author Response

“In the significance statement, the authors state that “ACh PMT neurons attenuate slow wave activity and stimulate cortical activation with theta and gamma that can be associated with either waking or PS depending upon the concurrent activity of other arousal systems”, which appears to be a conclusion that can be drawn from previous studies.”

Response: The conclusion that ACh PMT neurons attenuate slow wave activity and stimulate cortical activation may have been suggested by previous work but was not proven. Specifically, neither our previous in vivo electrophysiological unit recording studies (Boucetta et al., 2014), which were purely correlative, nor the recently published optogenetic (Van Dort et al., 2015) or chemogenetic (Kroeger et al., 2017) studies, which lack in vivo electrophysiological unit recordings, could reach this conclusion. In our current study, we bring forward for the first time proof of this with optogenetic stimulation of identified ACh unit firing that evokes, while being correlated with, specific alterations in EEG activity.

Revisions: We have changed the concluding sentence to bring out the significance of the study

better by referring to conclusions drawn in the Discussion that: “We conclude that by tonic or burst discharge during W and PS, ACh PMT neurons promote theta with gamma activities that are known to be important for memory along with other cortical processes during these states.”

Reviewer #1:

1. With regard to causal evidence for the role of cholinergic neurons in PS, the Reviewer states that “one study showed that optogenetic stimulation of cholinergic neurons increases entry to PS... (Van Dort et al., 2015).”

Response: As presented in our discussion, Van Dort et al. “stimulated the ACh neurons for prolonged periods (60, 80 and 120 s) and examined the probabilities of state change during long periods (30 s up to 3 min) following the photostimulation.” The authors stated that “REM sleep probability was significantly higher... for the 30-60 s beyond the stimulation”. Yet the authors do not know what the cholinergic neurons were doing during that period. Indeed, in our study, it appeared that the ACh neurons dramatically slowed or stopped firing upon cessation of the photostimulation, likely due to strong activation of K+ currents. Our analysis is therefore based upon the evoked discharge of the ACh neurons during the photostimulation in

relation to the concurrent EEG and state activity. Moreover, unlike mice used in our study, the

BAC transgenic mice used by Van Dort et al. contain ~50 extra copies of the VAChT gene, have

20-fold higher levels of VAChT mRNA in the striatum, have 6-fold higher levels of brainstem VAChT protein, release 3-times more ACh from the hippocampus and display a hypercholinergic phenotype (Kolisnyk et al., 2013; Crittenden et al., 2014), all of which calls into question the interpretation of findings derived from that model.

Revisions: We have added the above points to the Discussion (p. 36) concerning the interpretation by Van Dort et al. of their effects of photostimulation.

2. “Another study showed that chemogenetic activation of cholinergic neurons decreases delta activity during SWS without causing a change in the sleep state amount (Kroeger et al., J. Neurosci. 2017), suggesting that while cortical activation is commonly induced by these neurons, whether a state transition occurs depends on the stimulation protocol.”

Response: Using chemogenetic stimulation, Kroeger et al. have no idea what the cholinergic

neurons are doing during the period of their analysis. Moreover, in that publication, it is pointed out by the authors (in the Discussion!) that only 20% of the targeted cholinergic neurons were actually labeled with the DREADD receptor, which could also explain the 'minor role' they concluded the cholinergic neurons play.

Revisions: The above points have been added to the Discussion (p. 36).

3. “Moreover, in a previous study, the current authors beautifully showed that, based on an elegant juxtacellular recording technique, many cholinergic neurons in the PPT are wake-PS active with maximal activity during PS, further supporting a role in cortical activation and perhaps in PS induction/maintenance (Boucetta et al., 2014).”

Response: While we appreciate the Reviewer's complimentary summary of our previous juxtacellular recording study in rats, we submit that the results of that study were purely correlative, whereas the results of the current study provide for the first time proof of the role of ACh PMT neurons in stimulating theta and gamma activity by applying optogenetic stimulation together with juxtacellular recording and identification of the ACh PMT neurons. Revisions: We have elaborated in the Discussion (p. 34) upon the findings of our previous study in the rat (Boucetta et al., 2014) to provide a more complete description of those findings and how they relate to the current study.

4. “While the authors discuss in the abstract various downstream mechanisms involved, including local and/or more distant targets in the hypothalamus and thalamus, this is also not addressed.”

Response: A detailed description of the targets within the brainstem and diencephalon, as previously documented in our and others' chemical neuroanatomical, pharmacological and electrophysiological studies, is provided in the Discussion (p. 34) with respect to the multiple local and distant targets and circuits through which the ACh PMT neurons could generate theta from the brainstem, as we demonstrate here for the first time.

Revisions: Having added a more full description in the Discussion (p. 34) of the findings of our previous study in the rat (Boucetta et al., 2014), we now relate those findings to the current

findings concerning which cells were facilitated or attenuated in the LDT/SubLDT and PPT by the activation of the ACh neurons (see also below).

Reviewer #2 (Rationale for Significance Rating for Authors (Required)):

“This is an extremely detailed look at these neurons, which covers a lot of ground that has been covered in other studies. Although there is new information, what is here is not going to be

very surprising or enlightening.”

Response: As stated in our response to Reviewer #1, we believe that we have provided for the first time proof that discharge by the ACh PMT neurons can evoke forebrain theta with gamma by applying optogenetics together with juxtacellular recording and identification of the ACh PMT neurons. We go on to fully study this phenomenon revealed by optogenetic stimulation by examining the relationship of the ACh PMT neuronal discharge in relation to EEG activity during natural sleep-wake states in both the transgenic (TG) and wild type (WT) mice. We

realize that what comprises 4 different electrophysiological studies (employing either whole cell

or juxtacellular recording in 2 different labs), one applying optogenetics in vitro, one applying optogenetics in the urethane-anesthetized TG mice, one applying optogenetics in the unanesthetized head-fixed naturally sleeping-waking TG mice and one in the unanesthetized head-fixed naturally sleeping-waking WT mice “represents an extremely detailed look at these neurons”, which we believe is necessary to understand the effects of the optogenetic stimulation and the relationship of evoked changes in ACh unit firing together with EEG and state that occur during natural sleep-wake states. We were not seeking results that would be “surprising” but the reality of what is the complexity of the discharge of the PMT ACh neurons and the way in which they can modulate EEG activity and state.

Reviewer #2

1. “Sadly, the authors have systematically avoided their data (which must have been collected) on the non-cholinergic cells in these nuclei. Recent studies (e.g., Kroeger et al, 2017, which is barely mentioned here), have used optogenetics” [my bold] “to show that the cholinergic cells play a relatively minor role, mainly with respect to EEG frequency, while the glutamatergic neurons in the same cell groups drive wakefulness. A paper addressing their findings with both types of neurons would be much more interesting.”

Response: First, we presented in the Results and Discussion, data showing and discussing that “within the LDT/SubLDT or PPT, ...NonACh neurons were modulated by facilitation (&gt;70%) or attenuation (&lt;30%)”. We believe that these results are important when interpreting the photo- activation of the ACh PMT neurons. The Reviewer does not appear to be familiar with our previous study in rats employing juxtacellular recording and labeling of glutamate (Glu) and GABA as well as ACh neurons in the LDT/SubLDT/mPPT of rats (Boucetta et al., JN, 2014). In

the latter study, we showed that the Glu and GABA neurons therein are heterogeneous in their sleep-wake discharge profiles (as described below). In the current (very demanding) juxtacellular recording study of the ACh PMT neurons, we could not also study these diverse Glu and GABA cell groups and how they would be specifically affected by the ACh photostimulation.

Revisions: We have now mentioned in the Introduction (p. 6) the Kroeger et al. study along with the Van Dort et al. study (above). Having added a more full description in the Discussion (p. 34) of the findings of our previous study in the rat (Boucetta et al., 2014), we now relate those findings to the current findings concerning which cells were facilitated or attenuated in the LDT/SubLDT and PPT by the activation of the ACh neurons.

Secondly, with regard to the study by Kroeger et al., it employed chemogenetics, not optogenetics, which does not have the temporal resolution of optogenetics. As stated above to Reviewer #1, the in vivo discharge of the ACh neurons under the stimulation of the DREADD receptor is totally unknown. Moreover, the authors stated (in the Discussion) that only 20% of targeted cholinergic neurons were actually labeled with the DREADD receptor, which could also explain the 'minor role' they concluded the cholinergic neurons play.

Revisions: First, we have now added to the Discussion (p. 34) a full description of the Glu and GABA functional cell types revealed in our previous study (Boucetta et al., 2014) and how they might correspond to the neurons in the LDT/SubLDT and PPT which were found to be facilitated or attenuated by the photostimulation of the ACh neurons.

Second, we have added mention of the fact that only 20% of the ACh neurons expressed the DREADD receptor and most importantly that the in vivo discharge of the ACh neurons during the stimulation was not known (Discussion, p. 36)

2. “What is missing here is some perspective. The field was broken open recently by the groundbreaking observations by Kroeger et al., 2017, who found that the PMT ACh neurons when driven optogenetically” [my bold] “had minor effects on wake-sleep behavior, and mainly reduced delta activity in the EEG ... What they do NOT mention is that the glutamatergic neurons in the same region of the PMT actively drove waking states very potently.”

Response: In addition to erroneously referring to the ACh neurons being “driven optogenetically”, the Reviewer along with the authors of the study do not actually know how or whether the ACh neurons or the glutamatergic neurons were stimulated to fire in vivo by the DREADD. As already stated, only by in vivo single unit recording, as we have performed, can

the discharge of the neurons be known and the relationship of that discharge to the alterations of EEG activity and sleep-wake states be interpreted. We have actually employed and shown the effects of optogenetically driving the ACh PMT neurons and also study and consider very carefully exactly what that driving represents in relation to the natural discharge of the ACh PMT neurons in relation to the natural EEG activity and sleep-wake states.

Moreover, as mentioned (above) we previously recorded in vivo from identified glutamate (Glu)

and GABA neurons together with the ACh neurons in the LDT/SubLDT/mPPT of rats (Boucetta et al., 2014). As we state in the Discussion of our current manuscript, in contrast to the ACh neurons, the Glu and GABA neurons are heterogeneous in their profiles of discharge in relation to EEG/EMG and sleep-wake states. Indeed approximately half or more of Glu and GABA neurons discharge in association with cortical activation during both Wake and PS, like the ACh neurons. In fact, this (W/PS-max profile) is the same profile of discharge originally described by Steriade for neurons of the midbrain reticular formation of the ascending reticular activating system (Steriade et al., 1982) and would represent the profile of multiple neurotransmitter cell types, most numerously being Glu, participating in cortical activation during both W and PS. In the LDT/SubLDT/mPPT, we reported that ~30% of the Glu neurons and half of the GABA neurons actually discharged maximally during PS (as PS-max active) and minimally during W. Remaining, only ~20% of Glu neurons discharged maximally during Wake (as W-max active)! From these data, it is clear that using pan-stimulation, opto- or chemo-genetic, of these functionally diverse Glu and GABA cell populations in this PMT (and other) regions, is not valid for concluding what 'the' role of the Glu and GABA cell groups are!

Revisions: We have added the above points to the Discussion (p. 34 and 36).

In summary, although we discuss these optogenetic (Van Dort et al., 2015) and chemogenetic (Kroeger et al., 2017) studies, which were published before our (very long) optogenetic with juxtacellular recording and labeling study, we do not believe that they have proven the role of ACh PMT neurons in EEG or states in any way that should impede the publication of our detailed and thorough electrophysiological study.

References

Boucetta S, Cisse Y, Mainville L, Morales M, Jones BE (2014) Discharge profiles across the sleep- waking cycle of identified cholinergic, GABAergic, and glutamatergic neurons in the pontomesencephalic tegmentum of the rat. J Neurosci 34:4708-4727.

Crittenden JR, Lacey CJ, Lee T, Bowden HA, Graybiel AM (2014) Severe drug-induced repetitive

behaviors and striatal overexpression of VAChT in ChAT-ChR2-EYFP BAC transgenic mice. Front Neural Circuits 8:57.

Kolisnyk B, Guzman MS, Raulic S, Fan J, Magalhaes AC, Feng G, Gros R, Prado VF, Prado MA (2013) ChAT-ChR2-EYFP mice have enhanced motor endurance but show deficits in attention and several additional cognitive domains. J Neurosci 33:10427-10438.

Kroeger D, Ferrari LL, Petit G, Mahoney CE, Fuller PM, Arrigoni E, Scammell TE (2017)

Cholinergic, Glutamatergic, and GABAergic Neurons of the Pedunculopontine Tegmental

Nucleus Have Distinct Effects on Sleep/Wake Behavior in Mice. J Neurosci 37:1352-1366. Steriade M, Oakson G, Ropert N (1982) Firing rates and patterns of midbrain reticular neurons

during steady and transitional states of the sleep-waking cycle. Exp Brain Res 46:37-51.

Van Dart CJ, Zachs DP, Kenny JD, Zheng S, Goldblum RR, Gelwan NA, Ramos DM, Nolan MA, Wang K, Weng FJ, Lin V, Wilson MA, Brown EN (2015) Optogenetic activation of cholinergic neurons in the PPT or LDT induces REM sleep. Proc Natl Acad Sci US A

112:584-589.
